# Drivers of HIV treatment interruption: Early findings from community-led monitoring program in Haiti

**DOI:** 10.1371/journal.pone.0295023

**Published:** 2023-12-05

**Authors:** Soeurette Policar, Alana Sharp, Joanne Isidor Hyppolite, Gérald Marie Alfred, Eva Steide, Leïnadine Lucien, Naiké Ledan, Matthew Kavanagh

**Affiliations:** 1 Organisation de développement et de lutte contre la pauvreté (ODELPA), Delmas, Haiti; 2 Center for Global Health Policy and Politics, O’Neill Institute for National and Global Health Law, Georgetown Medical Center, Georgetown University, Washington, DC, United States of America; 3 Housing Works, Port-au-Prince, Haiti; 4 Action citoyenne pour l’égalité sociale en Haïti (ACESH), Marchand Dessalines, Haiti; 5 Health GAP, New Orleans, LA, United States of America; 6 Department of Global Health, School of Health, Georgetown University, Washington, DC, United States of America; University of Zimbabwe Faculty of Medicine: University of Zimbabwe College of Health Sciences, ZIMBABWE

## Abstract

**Background:**

Failure to retain people living with HIV (PLHIV) in care remains a significant barrier to achieving epidemic control in Haiti, with as many as 30% lost from care within one year of starting treatment. Community-led monitoring (CLM) is an emerging approach of improving healthcare and accountability to service users, through a cycle of monitoring and advocacy. In 2020, a CLM program was launched in Haiti to identify barriers to retention and advocating for better health services.

**Methods:**

Data from the community-led monitoring program in Haiti were analyzed, from a sample of 65 healthcare facilities in the Nord, Artibonite, and Ouest departments collected from April 2021 to February 2022. Qualitative data from six community-based focus groups and 45 semi-structured individual interviews were analyzed.

**Results:**

Confidentiality and stigmatization emerged as barriers to care, particularly due to the separation of PLHIV from other patients in view of community members. To avoid identification, patients described traveling long distances, with the reimbursement of transportation costs described as being insufficient or unavailable. Costs of non-HIV clinical services were a frequent concern and respondents described a need for clinics to provide food during all patient visits. Stock-outs were a regular challenge; by contrast, treatment literacy did not emerge as a major barrier to retention.

**Conclusions:**

These findings represent the first instance, to our knowledge, of original data from a community-led monitoring program being published in any country. These findings suggest that improving treatment retention for PLHIV is dependent on improving the acceptability and affordability of healthcare services. Ensuring confidentiality is critical, particularly where stigma is high. Retention could be improved by systematically strengthening patient confidentiality protections throughout the healthcare system, providing patients with sufficient travel compensation and other incentives, and delivering wraparound services provided for free. Addressing these challenges will require ongoing advocacy for community-developed recommendations and solutions.

## Introduction

Seven years remain to achieve the UNAIDS 95-95-95 Fast-Track targets [1]. In Haiti, only 72% of all people living with HIV (PLHIV) have achieved viral suppression [2], well below the global target of 86%. In recent years, an estimated 18.7% to 30.1% of all PLHIV receiving antiretroviral therapy dropped out of care within one year [3, 4]. This failure to successfully treat and retain PLHIV in care are key barriers to epidemic control in Haiti.

In 2020, Haitian community-based organizations of PLHIV, key populations (KP), and adolescent girls and young women launched a community-led monitoring (CLM) program to identify and address barriers to treatment retention and access. Building on a growing trend of facility monitoring and community responses [5], CLM is a tool for strengthening accountability of donors and governments to service users, by empowering local community organizations to gather data on healthcare quality and access, develop solutions to barriers to care, and advocate directly to governments, donors, and other stakeholders [6]. In contrast to other quality improvement strategies, the CLM model acknowledges that transparency cannot itself improve healthcare quality, particularly in a donor-monopolized HIV response where patients are not empowered or able to act on clinic metrics. As such, CLM uses community-owned data to strengthen lines of direct feedback between civil society and governments, donors, and other decision-makers. Additionally, a key differentiating factor between CLM and other donor-supported monitoring mechanisms is the leadership and ownership of all programmatic and governance aspects by local, community members and civil society organizations.

These early findings from the CLM program present an empirical assessment of the drivers of high loss to follow-up (LTFU) rates in Haiti. To our knowledge, this analysis is the first presentation of the findings from a community-led monitoring program of HIV services in the peer-reviewed literature.

## Methods

### Community-led monitoring data

All data were gathered by *l’Observatoire Communautaire des services VIH* [The Community HIV Services Observatory] (OCSEVIH). OCSEVIH is a consortium of local, community-based organizations partnering to lead the CLM. Quantitative data were gathered from the public data dashboard of the CLM program [7] and qualitative data were shared with the investigators by the CLM program.

The CLM program gathered quantitative survey data in healthcare facilities, in order to measure the availability of services and the experiences of clinic clients. These data were complemented by interviews conducted in neighboring communities, which provided the experiences of PLHIV and KP who are LTFU or not currently receiving facility-based care.

Survey instruments had been designed by the CLM program during a week-long workshop with civil society organizations, associations of PLHIV and KP, and with technical support from an academic research team. Three survey tools were designed: one for the community monitors to record their own observations of clinic operations, one to survey clinic patients, and one for facility managers. Indicators were selected to gather data on key barriers and enablers of healthcare quality and access, with the aim of gathering data for advocacy activities. In line with PEPFAR guidance [8], indicators were intentionally non-overlapping with those in PEPFAR’s Monitoring, Evaluation and Reporting (MER) framework.

Data collection was performed by a team of 11 community monitors (CM), who were trained PLHIV and/or KP from the community. Survey responses were collected and stored using Dimagi’s CommCare application [9] on Wi-Fi-enabled tablets. All survey data are publicly available online [7]. All data were anonymous, and as such the authors did not have access to any personally-identifiable information.

Data were gathered in 65 health facilities located in 16 *arrondissements* [districts] in the North, West, and Artibonite departments (**Table 1**). Data collection occurred in two phases: in the first phase, surveys were conducted in 41 facilities from April to June 2021, and in the second phase an additional 25 facilities were surveyed from December 2021 to February 2022. Of the sites sampled, 39 were primary healthcare clinics that delivered HIV services, while two were HIV-specific clinics. Facility visits were announced to clinic management by telephone to ensure that CM would be permitted to enter. The data analysis was conducted from May to July 2022.

**Table 1:** Survey, focus group, and individual interview respondent characteristics.

Characteristic	Patient surveys	Focus groups	Individual Interviews
	**N = 1,677**	**N = 6**	**N = 45**
*Département*			
** Artibonite**	499 (29.8%)	2 (33.3%)	15 (33.3%)
** North**	536 (32.0%)	2 (33.3%)	15 (33.3%)
** West**	640 (38.2%)	2 (33.3%)	15 (33.3%)
** Total**	1,677 (100.0%)	6 (100.0%)	45 (100.0%)
**Gender**			
** Female**	1,160 (69.2%)	NC	NC
** Male**	514 (30.6%)	NC	NC
** Transgender**	1 (0.1%)	NC	NC
** Total**	1,677 (100.0%)	NC	NC
**Age**			
** >18 years**	82 (4.9%)	NC	NC
** 18–25 years**	237 (14.1%)	NC	NC
** 26–39 years**	614 (36.6%)	NC	NC
** 40+**	609 (36.3%)	NC	NC
** No response**	128 (7.6%)	NC	NC
** Don’t know**	7 (0.4%)	NC	NC
** Total**	1,677 (100.0%)	NC	NC
**PLHIV**			
** Yes**	1,182 (70.5%)	6 (100%)	45 (100%)
** No**	495 (29.5%)	0 (0%)	0 (0%)
** Total**	1,677 (100.0%)	6 (100%)	45 (100%)
Key population (*among PLHIV*)			
** Yes**	183 (15.5%)	2 (33.3%)	36 (80%)
** No**	989 (83.7%)	4 (66.7%)	9 (20%)
** Prefer not to answer**	10 (0.8%)	0 (0%)	0 (0%)
** Total**	1,182 (100.0%)	6 (100%)	45 (100%)

PLHIV = people living with HIV, MSM = men who have sex with men, SW = sex worker, NC = not collected.

Survey participants were selected from the clients in the clinic or among those waiting outside, with monitors intentionally seeking a balanced sample with regard to gender and age. After an initial set of generic questions, respondents’ HIV status was screened and those with HIV received additional questions. This strategy was used to build rapport before querying more sensitive topics.

Qualitative data were gathered through semi-structured individual and focus group interviews using interview guides. Two populations were targeted for interviews: PLHIV and KP (men who have sex with men, sex workers, and transgender people) and participants were recruited through grassroots civil society organizations (**Table 1**). Respondents were invited to join either an individual interview or a focus group on the basis of their level of comfort sharing information in a group setting.

Six focus groups and 45 individual interviews were conducted in the community using a convenience sample, with participants recruited based on referrals from the PLHIV, LGBT, and sex worker sectors. Additionally, respondents were recruited through the surveys conducted in facilities. Interviews were conducted in Creole or French, with the audio recordings transcribed and translated into English for analysis. This study was approved by the Georgetown University institutional review board. Informed consent was obtained from all participants, electronically for survey respondents and orally for interview and focus group participants.

### Data analysis

Using the combination of quantitative survey and findings from the interviews, the CLM program assesses drivers of LTFU in HIV clinical services. Enablers and barriers to care were described along a continuum of four factors driving health utilization and retention in care: availability of services and health-seeking behavior, acceptability of services, accessibility of services, and affordability [10].

We note that the indicators developed by the CLM program are not developed by medical professionals or clinicians, but are created by PLHIV, KPs, and other community members who are users of services. As such, a strong focus on patient perception of quality underlies the dataset, rather than on the delivery of medically-appropriate clinical interventions [11].

Survey data from the observation tool, the patient survey tool, and the facility manager tool were extracted from CommCare. Data were aggregated across both cycles of data collection, noting that data were only collected from each facility one time in this period. Where applicable, the response choice “prefer not to answer” was excluded from the analysis.

Qualitative data were analyzed with the aim of identifying barriers to, and enablers of, access to facility-based and community-based healthcare. Since interviews were conducted outside of healthcare facilities, the findings from this population were intended to complement the facility-based data with in-depth, qualitative data about barriers and challenges accessing care.

Data from the interviews were organized into discrete units consisting of one question and one response (hereafter, “chunks”). Chunks were first reviewed using a holistic approach, in which each chunk was reviewed and assigned to one or more themes [12, 13]. Themes were developed through a combination of deductive and inductive approaches, in which a first set of themes were developed *a priori*, corresponding to the themes in the interview guides and survey tools. During analysis, a second set of additional themes emerging from the data were identified using an inductive approach.

A second phase of sub-coding was conducted, in which chunks were inductively coded with more granular themes. Chunks were then tabulated across each thematic category.

Throughout the results, survey data are indicated as percentages, while findings from qualitative data are only quantified through the indication of number of respondents discussing each theme. Detailed findings from the qualitative analysis are found in Appendix.

## Results

### Availability of services and health-seeking behavior: Treatment literacy is high, stock-outs barrier to care

Two barriers to treatment retention were measured, to assess whether stock-outs and shortages of medications (supply-side) or a lack of treatment literacy among patients (demand-side) are potential contributors to loss to follow-up.

**Fig 1:**
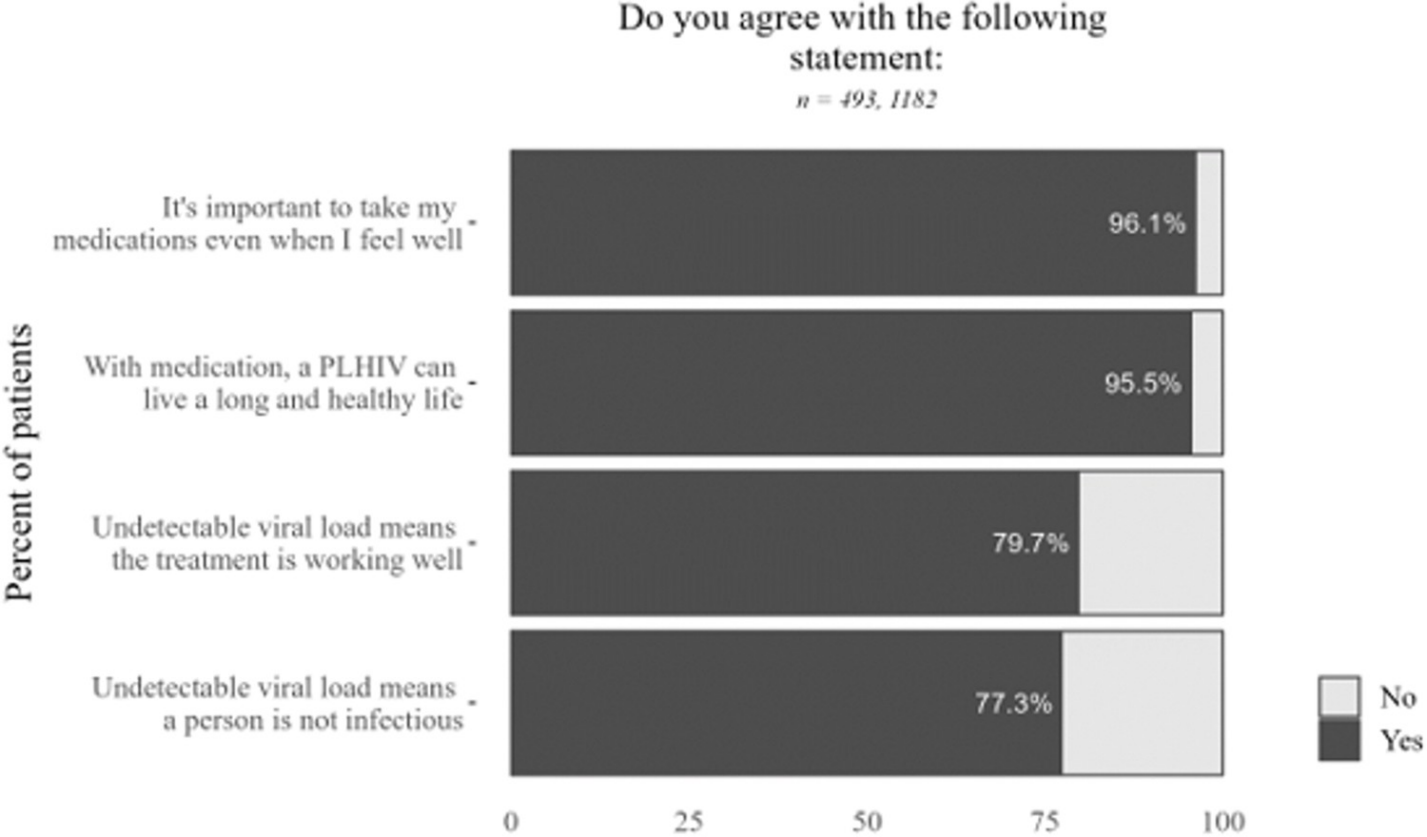
Survey data on treatment literacy and awareness of treatment as prevention. Undetectable = untransmittable, or U = U. Sample size varies due to skip logic and questions not asked in all rounds of data collection.

According to survey data, treatment literacy was high among PLHIV surveyed in clinics. Nearly all respondents were aware of the importance of taking their medications (96.1%) and that PLHIV can live a long and healthy life with medication (95.5%) (**Fig 1**).

Both clinic and community respondents identified a desire for more clinical counseling, which was described as an important motivator for remaining in care. A small number of interviewed respondents described not believing their diagnoses, misunderstanding HIV being a lifelong infection, or only taking medications when symptomatic. However, lack of treatment literacy did not emerge as a significant theme or driver of patients falling out of care.

“*Now I have a lot of information*. *HIV can’t kill me*, *I can die of another disease […] Basically*, *we are told about the virus*, *if we take our medication*, *the disease will not progress*, *and one day we will be undetectable*.*” (KP PLHIV from Ouest)*

Stock-outs of antiretroviral (ARV) drugs emerged as a driver of treatment interruption. According to both facility manager and patients, PLHIV were regularly sent home without medications (8% to 12% reported patients leaving without drugs due to stock-outs in the preceding two months, respectively). Another 13% of clinic managers described giving shorter prescriptions during shortages, and five interviewees described receiving expired or soon-to-be expired medications due to limited stock (results not shown).

**Fig 2:**
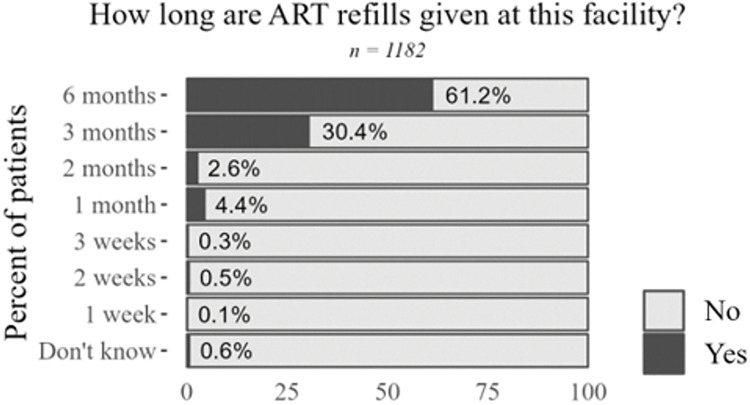
Survey data on antiretroviral refill length. Sample size varies due to skip logic and questions not asked in all rounds of data collection.

Prescription length did not emerge as a barrier to care, with nearly all respondents receiving ARV refills for at least three months at a time (91.6%) (**Fig 2**). However, long prescriptions were not always described in positive terms; in some cases, large stocks of pills created risks of disclosure to other members of the household.

“*When I was offered [medicines]*, *they told me to take a test and offered it for a period of 6 months*. *I didn’t want it for so long*. *I didn’t want to raise suspicions at home*.*” (KP PLHIV from Artibonite)*

Stock-outs of commodities other than antiretrovirals were common, with 75% of clinic managers reporting at least one stock-out in the past two months. More than half of facility managers (58%) reported that lubricants had been unavailable. Other products that faced stock-outs were drugs for opportunistic infections, iron and vitamins, and female/internal condoms (results not shown).

Notably, in some cases services existed but were not well communicated to patients, primarily around access to support groups and PrEP (results not shown). Survey data indicated that participants of support groups receive counseling on medication side effects (74%), treatment and adherence (54%), and information on different medication options (42%). In addition to the provision of clinical information, seven interviewed respondents described benefiting from a sense of community and emotional support in the groups.

“*When we get together at the support group*, *it’s like we are family*. *We share ideas together*, *you feel liberated*, *we have the same problem and we support each other*.*” (PLHIV from Nord)*

Support groups were reportedly available in nearly all clinics, with 94% of facility managers indicating that support groups were available at their clinics. Yet just 55% of patients surveyed were aware of any such groups. Seven participants described in interviews that support groups were either unavailable or had been discontinued.

Awareness of PrEP was similarly inconsistent. While 46% of facility managers surveyed reported that PrEP was available at their facility, only 25% of clients reported having ever heard of the medication (results not shown). In interviews, 25 respondents were aware of PrEP, while 15 were either unaware of PrEP or had never received counseling on PrEP. While respondents living with HIV would not be candidates for PrEP themselves, this gap in clinical counseling and access to information indicates a barrier to prevention services for people in serodiscordant partnerships.

### Acceptability of services: Fear of disclosure and confidentiality violations are common

Disclosure of HIV status and confidentiality violations while receiving care in a clinic emerged as a significant concern, with 23 interview respondents described a fear of being seen by other clients or community members while in the clinic and 18 reporting the practice of clinical staff disclosing patients’ HIV status without consent.

**Fig 3:**
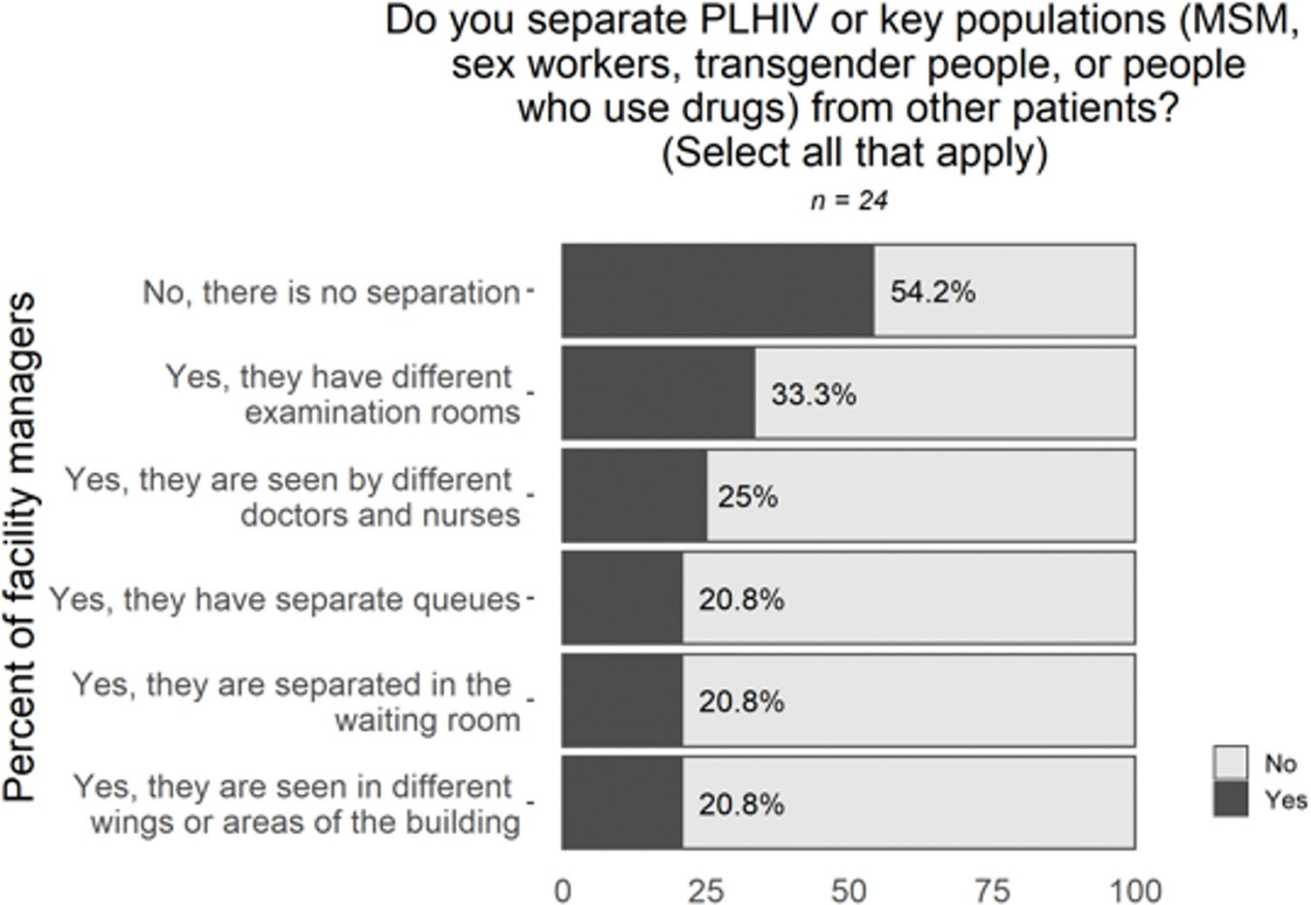
Survey data on privacy and patient confidentiality. Sample size varies due to skip logic and questions not asked in all rounds of data collection.

The practice of separating patients was cited by 18 respondents as being a primary driver of stigma and privacy violations. According to facility managers, 46% of clinics implemented some sort of separation of PLHIV from other patients, primarily in the form of different examination rooms (in 33% of clinics) and different doctors and nurses (25%) (**Fig 3**) Four health centers reported all five forms of separation: PLHIV-only waiting rooms, separate queues for PLHIV, providing HIV care in separate areas in the building, separate clinical staff for PLHIV, and reserving specific examination rooms for patients with HIV.

Additional patient concerns included being asked to wait on dedicated benches for PLHIV or KP patients, signs on buildings or in hallways identifying HIV clinics, receiving colored appointment cards visibly identifying their HIV status or sexual orientation, counseling in shared rooms or public spaces, and indiscreet dispensing in transparent or noisy pill bottles.

“*There are some places where they receive patients in threes*. *I could explain something personal to the doctor*, *but I can’t because of the two other people who are close by*. *Just because we are all PLHIV patients doesn’t mean that we don’t have a private life*, *that everyone has the same problem*.*” (KP PLHIV from Ouest)*

Respondents also described nurses, physicians, and other staff disclosing clients’ HIV status either by discussing personal information in shared spaces, sharing information outside of the clinic to the client’s community, or by directly providing medical information with the client’s friends or family members.

“*As soon as you test positive for HIV*, *the nurse will divulge the information to anyone who crosses her path*, *to your neighborhood*, *to your friends if they know them*. *[…] Even in the hospital building*, *[…] at the very beginning of the entrance there is an arrow indicating "HIV patient"*. *This is the space that is made for us*. *As soon as someone who frequents the hospital sees you going in that direction*, *you are already identified*. *And the news is spreading throughout the city*.*” (KP PLHIV from Ouest)*

The consequences of disclosure were described as intense stigmatization, with 20 respondents described experiencing discrimination, bullying, and humiliation and 19 described being ostracized by their communities for their HIV status or sexual orientation. Others reported loss of employment, loss of access to education, homelessness or needing to move, and threats of violence.

While patients surveyed in facilities generally described staff as being professional (84%) and welcoming and friendly (94.5%), respondents outside of clinics shared negative views (results not shown). 28 community respondents described rude or unprofessional staff behavior. Twenty-seven respondents described stigmatization by clinical staff due to HIV status or sexual orientation.

“*At the center to be touched*, *the staff wears gloves*, *they treat you like a dangerous species*. *[…] Sometimes the staff treated us with disdain and even insulted us because of our seropositive status*. *We were humiliated once they found out about our condition*.*” (PLHIV from Nord)*

### Accessibility of services: Patients seek improved care through transfers to distant clinics

**Fig 4:**
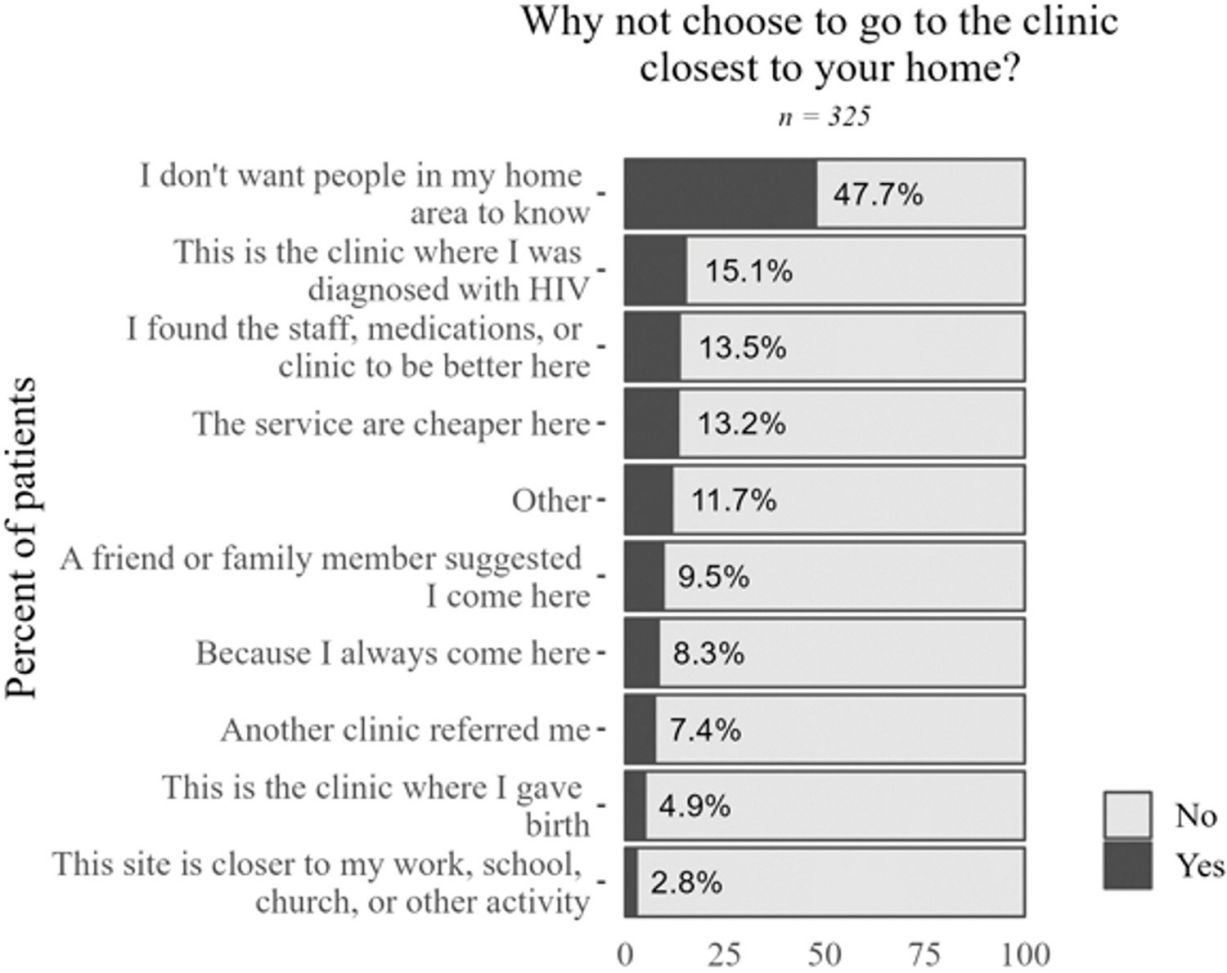
Survey data on geographic accessibility of clinics and travel. Sample size varies due to skip logic and questions not asked in all rounds of data collection.

Driven by concerns about being seen in a clinic, many patients intentionally traveled to faraway clinics where they will not be recognized (**Fig 4**). More than 60% of clients surveyed in clinics described traveling long distances to visit clinics, with 57% of respondents traveling one hour or longer despite most being aware of other clinics nearer to them.

Transferring to different clinics was often the only recourse available to patients experiencing poor service delivery. Yet interview participants described transfer requests being denied or facing onerous bureaucratic challenges, with patients choosing to move between clinics without institutional approval.

“*Everyone needs numbers*, *[the clinic] wants to justify providing the service to a lot of PLHIV*, *they are not going to [agree to a transfer]*.*” (KP PLHIV from Ouest)*

Delivery of medications was described by interviewees as an important tool to protect patient privacy at the clinic, and to reduce transportation costs and clinic wait times. Yet 47% of surveyed patients chose not to have medications delivered due to privacy concerns (results not shown). The fear of HIV status disclosure during delivery was a key theme in interviews, with respondents describing indiscreet delivery practices or disclosure by association with a delivery person whose role is known to the community.

### Affordability: Insufficient travel compensation and user fees create financial burden

**Fig 5:**
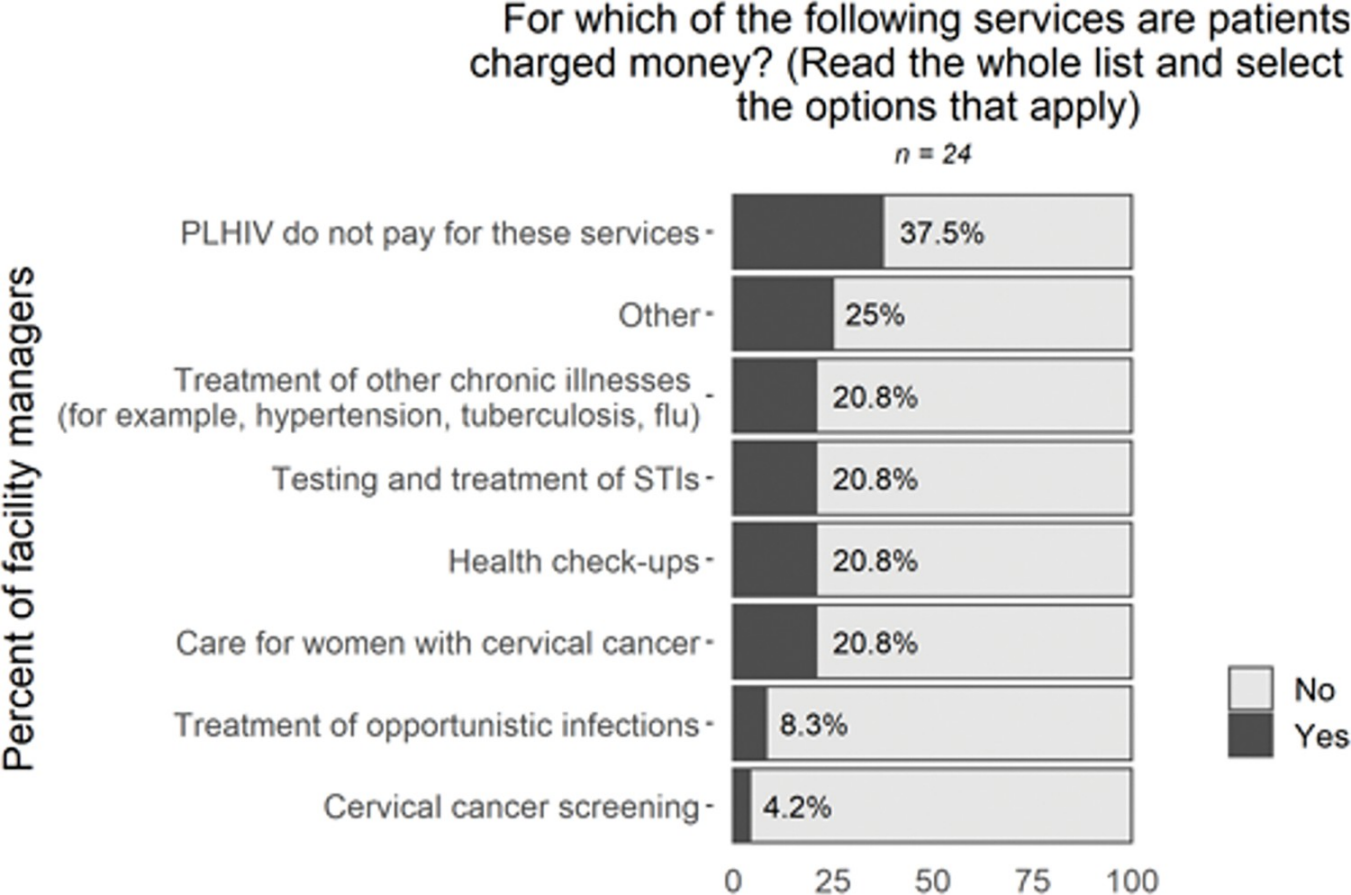
Survey data on costs and incentives. Sample size varies due to skip logic and questions not asked in all rounds of data collection.

Fees for services were identified as a barrier to care, with users describing seeking HIV services but facing costs for other medical services. While HIV care is intended to be available without user fees in Haiti [14], seven interviewees reported being charged for wraparound services and other routine healthcare. Facility managers indicated that in 21% of clinics PLHIV are charged fees for treating other chronic illnesses (such as hypertension or tuberculosis), testing and treatment for sexually-transmitted infections, health check-ups, and cervical cancer services (**Fig 5**).

Transportation costs were also cited as a barrier, particularly given the common practice of receiving care far away from their homes due to privacy concerns. Travel costs were frequently reimbursed by clinics, with 95% of patients surveyed receiving some transport fees (results not shown). Yet these subsidies were described as being either insufficient, inconsistent, or only being available hours after the clinical appointment concluded. Eight interview and focus group respondents explicitly described ending treatment due to transit costs.

“*When I ask for money for transportation fee*, *they said it would be available at 2 o’clock*. *I have to beg in the streets so that someone gives me 50 gourdes to go home*.*” (PLHIV from Nord)*

Twelve participants described the need for food and economic support, with eight being unable to take medication due to food insecurity, often due to nausea and dizziness when taken on an empty stomach. Lack of food was described as a driver of treatment discontinuation and interruption.

“I *get short of breath and sometimes I get dizzy if I don’t eat*, *and the pills are big*, *you have to eat*. *Before*, *[the clinic] used to accompany the medication with a small food kit*, *we don’t do that anymore*. *If a person who doesn’t work*, *who doesn’t earn anything*, *has to take these drugs*, *they have to find something to eat*.*” (KP PLHIV from Ouest)*

## Discussion

These findings from the Haitian CLM program provide an empirical assessment of the key drivers of low retention in HIV care, using data gathered by and for service users, communities, and advocates. In addition to substantiating prior research findings, these data are a novel example of impacted communities in Haiti gathering and using their own empirical data to identify gaps in service delivery. The indicators, designed by PLHIV and KP users of healthcare in Haiti, provide a hyper-local and granular view of facility-level challenges, while additionally gathering first-person narratives from community members living with HIV. This model of adaptable, near real-time, community-owned, and advocacy-oriented monitoring and evaluation holds potential for improving service delivery in the public health system. This analysis highlights the value of CLM in developing and implementing data collection plans to identify key gaps in HIV service delivery.

This assessment of the barriers and enablers of HIV care for Haitians reveals important drivers of LTFU. Survey data and interviews conducted in and out of clinics reveal relatively high levels of treatment literacy, although patients experienced inconsistent availability of HIV medications.

By contrast, concerns around patient confidentiality and stigmatization emerged as core barriers to retention. Disclosure of HIV status and/or sexual orientation and staff attitudes were recurrently described as disincentivizing patients from visiting health clinics and receiving care in their own communities. In particular, the practice of clinics separating PLHIV and KP patients from other patient populations was described as being stigmatizing and leading to disclosures of personal information to other patients.

Travel to clinics was a particularly large barrier for those intentionally traveling to avoid stigma or identification. Although transportation costs should be reimbursed by clinics, these payments were often insufficient or inconsistent. Although ARV delivery can reduce the costs and inconvenience of travel, some respondents reported being fearful of disclosures of HIV status or KP status during delivery.

Finally, concerns about the costs of non-HIV services in clinics was a recurrent theme, as well as the need to provide food, economic support, and other incentives during patient visits. This finding builds on prior research in Haiti suggesting that high transportation costs are significantly associated with greater likelihood of being lost to follow-up [15]. In a context where half of the country is undernourished [16] and living in poverty [17], and where PLHIV and KPs face significant stigma and barriers to employment, the provision of food is an important enabler of retention in care.

These findings are consistent with epidemiological estimates suggesting that while nearly 98% of PLHIV who know their status are on treatment, just 87% of those on treatment are virally suppressed [18]. This analysis builds on previous research in Haiti, which finds intense stigma experienced by PLHIV [19] and key populations and their partners [20], the need for strong patient support and clinician training [21], and strong correlations between stigma and treatment discontinuation [22]. Most recently, a series of focus group discussions with patients and healthcare workers in Port-au-Prince and Cap-Haïtien identified similar themes including deeply-held concerns about privacy violations, the lack of a “welcoming” atmosphere for clients, and insufficient nutritional and financial support [23].

Achieving the 95-95-95 targets in Haiti will require efforts from the *Programme National de Lutte contre le Sida* [National AIDS Control Program] (PNLS), PEPFAR and its implementing partners, Global Fund, and other stakeholders to improve the acceptability, availability, and affordability of clinical care. Key opportunities include designing clinical spaces so that the HIV status of PLHIV is not identifiable to other patients (e.g. integrated waiting rooms), implementing stigma reduction strategies (such as staff training and sensitization, welcoming environments, and ART delivery) ensuring that reimbursements of travel costs and other incentives are adjusted for the patient’s origin and for inflation, and conducting targeted outreach to increase awareness of services available to PLHIV and KP. Implementing quality improvement strategies should be implemented in partnership with PLHIV and KP networks, in order to ensure that interventions address these barriers to care. Existing efforts by PEPFAR and PNLS to de-duplicate patient records through biometric coding [24] are also intended to address the underlying drivers of doctor shopping, by ensuring that the quality, availability, and appropriateness of care is equivalent across all clinics; although care must be taken to ensure absolute confidentiality and protection of patients’ privacy.

This analysis reports on data gathered during community-led monitoring, which has a fundamental goal of data-informed advocacy rather than primarily empirical health service research. Given the project goals, randomization of the sites selected for monitoring was neither appropriate nor feasible, which limits generalizability of the empirical findings. Additionally, since the sampling of the participants was not formally randomized, these findings may not represent the full breadth of PLHIV and KP perspectives; for instance, since the plurality of patients surveyed were adult women that did not identify as KP, the findings from survey data may not adequately represent the experiences of young people, men, and key populations. Furthermore, while all interviewed respondents were PLHIV, not all clients in clinics self-identified as such; similarly, the differing methods used in the two settings (quantitative surveys versus more open-ended interviews) may produce non-representative or incomplete results.

The results nonetheless provide important and valid insights, particularly given the mixed-methods approach using quantitative and qualitative data sources. This analysis further acts as a proof-of-concept for the CLM model in Haiti, in which a community-driven process of evaluating gaps and gathering data has successfully identified key barriers to continuity in care. In a context where the quality of care in public facilities is highly variable [25], the creation of a ‘living’ facility-level database on the quality of care is a powerful tool for community activists. Indeed, the steps that must be taken to achieve the 95-95-95 targets are known; what the HIV response needs are powerful tools to enable community actors to demand those steps be taken. The CLM program is currently using these data to engage with decision-makers in an effort to enact change.

## Supporting information

S1 Table Barriers and enablers related to availability of services and health-seeking behavior. (DOCX)

S2 Table Barriers and enablers related to acceptability of services. (DOCX)

S3 Table Barriers and enablers related to accessibility of services. (DOCX)

S4 Table Barriers and enablers related to affordability. (DOCX)

S5 Table Survey data used in the analysis. (CSV)

S6 Table Geographic indicators used in the analysis. (CSV)

S7 Table Definitions of survey indicators. (CSV)

S1 Fig Survey data on stock-outs and shortages. Sample size varies due to skip logic and questions not asked in all rounds of data collection.
(DOCX)

S2 Fig Survey data on availability of, and knowledge of, support groups and PrEP. Sample size varies due to skip logic and questions not asked in all rounds of data collection.
(DOCX)

S3 Fig Survey data on privacy and patient confidentiality. Sample size varies due to skip logic and questions not asked in all rounds of data collection.
(DOCX)

S4 Fig Survey data on geographic accessibility of clinics and travel. Sample size varies due to skip logic and questions not asked in all rounds of data collection.
(DOCX)

S5 Fig Survey data on costs and incentives. Sample size varies due to skip logic and questions not asked in all rounds of data collection.
(DOCX)
