# Supplementary material for: Drivers of HIV treatment interruption: Early findings from community-led monitoring program in Haiti
Source: PLoS One. 2023 Dec 5;18(12):e0295023. doi: 10.1371/journal.pone.0295023 (PMC10697516; doi:10.1371/journal.pone.0295023)
Supplement: S1 Table — (DOCX) [file pone.0295023.s001.docx]

| **Type** | **Parent Category** | **Child Category** | **N** | **Example quotes** |
| --- | --- | --- | --- | --- |
| Barrier | PrEP | Unaware of PrEP/did not receive counseling | 15 | “This is the first time I heard this word” |
|  | Treatment Literacy | Needing more/any counseling/education | 15 | “No, they haven’t explained much about our medicine. […] Generally, the client doesn’t know which medicine they’re taking.” |
|  | Support Groups | Groups currently or no longer available | 7 | “I wish God would help them to find other medicines to give us and I wish they would start again with the support groups.” |
|  | Treatment Interruptions | Forgetting to take medications consistently | 7 | “The difficulty is that every morning you get up and think about what you have and there is one thing or medicine you have to take, it becomes a burden sometimes” |
|  | Psychosocial support | No counseling available or hard to access | 6 | “I would like to have more access to the psychologist's service because I don't feel like I'm at my best, I'm not normal like I used to be. At the beginning I was being supervised a lot, but I thought that it would continue until I was in good shape.” |
|  | Condoms and Lubricants | Staff not proactively offering / client not asking | 5 | “They don't offer me, I don't ask.” |
|  | Treatment Interruptions | Not knowing to take medication consistently | 4 | “I didn't really understand the disease. I thought I was going to die. I was thinking about my 2 kids and I was thinking, there's no reason for me to be on medication because I'm going to die soon even if I take it.” |
|  | Treatment Literacy | Not believing diagnosis or medical advice | 4 | “A lot of people don’t really believe their diagnoses.” |
|  | Diagnosis | Depression | 4 | “After I got the [diagnosis], I didn't go home. I spent two days at a friend's house, I couldn't eat or drink. I thought I was going to die right away.” |
|  | Support Groups | Insufficient pay/professionalization for peer navigators | 3 | “The peers lack means as well. The fees that they provide to the peers aren’t sufficient. We often have to use that money to do the work and it’s all because they don’t take the peers seriously enough. They don’t value our profession” |
|  | Diagnosis | Failure to initiate treatment after diagnosis | 3 | “But when I was pregnant, that’s when I found out I had the virus. I didn’t get any service, they just told me I was sick when I went to give birth.” |
|  | Diagnosis | Negative staff experience during diagnosis | 3 | “And the doctor told me the news very savagely without any mercy: ‘Madam, you have AIDS.’” |
|  | Treatment Interruptions | Stopping treatment because of depression | 3 | “I chose to stop taking my medication. I stopped for two months because I wanted to die.” |
|  | Psychosocial support | Difficulties accessing psychosocial support | 2 | “Some psychologists who work in these programs are just there on paper. […] Some clients go to the hospital and they don’t even know there’s a psychologist there.” |
|  | Treatment Literacy | Only taking medications when feeling ill | 2 | “I didn't feel ready, I wasn't sick. I was wondering about the veracity of the result. Then I had symptoms. I didn't feel physically fit, so I decided to take the medication.” |
|  | Treatment Literacy | Counseling from voodoo priests | 2 | “They go to a voodoo priest who gives a counter-diagnostis and the patient never returns for medical treatment.” |
|  | Support Groups | Not interested in groups | 1 | “I was invited to take part in a group, but I wasn't interested. […] But I don't like to walk, I don't like to go out.” |
| Enabler | Treatment Literacy | Receiving information from clinicians or support groups | 29 | “Basically, we are told about the virus, if we take our medication, the disease will not progress, and one day we will be undetectable” |
|  | PrEP | Aware of PrEP / received counseling | 25 | “Yes, PrEP is when someone does not have the sickness, to stay healthy when you have sex with someone who is infected” |
|  | Diagnosis | Positive staff experience during diagnosis | 15 | “I got my HIV results a year ago. […] They spoke to me calmly. I broke down, and I got really scared. Then they convinced me that life wasn’t over, that I would just need to take my medicine.” |
|  | Psychosocial support | Received or offered counseling – neutral | 14 | “Yes, [the clinic’s] psychologist supports me. But my biggest psychologists are the people back home, my parents.” |
|  | Treatment Interruptions | Reasons to stay in care: encouragement from facility and support groups | 12 | “I had stopped the treatment in 2016, it was only in 2020 that I started again. The reason I started again was because some agents came to my house and encouraged me to do the treatment because I didn't want to accept my status. The agent lady called me once, she accompanied me to do the test and I found out my status, I cried, she comforted me and she encouraged me to come back.” |
|  | Psychosocial support | Received counseling – positive experience | 9 | “One of the major reasons why I keep getting my treatment is thanks to the psychosocial help I received.” |
|  | Viral Load | Knowledge of viral load, U=U | 8 | “My viral load is low because I take my medicine regularly.” |
|  | Support Groups | Helpful services | 7 | “The support group is important because the information they give helps us, are good advice, on how to protect ourselves.” |
|  | Support Groups | Feeling of community and emotional support | 7 | “When we get together at the support group, it’s like we are family. We share ideas together, you feel liberated, we have the same problem and we support each other.” |
|  | Diagnosis | Successful initiation of treatment after diagnosis | 4 | “Finally, they told me that I am a PLHIV and I was enrolled the same day. They took me to [the clinic] and I started my treatment the same day.” |
|  | Psychosocial support | Ideas for how to do better | 3 | “What if we use a different tactic, we ask the on-site psychologist to deliver the transportation fees? This way we would be certain that all the patients see him/her before leaving.” |
|  | Treatment Interruptions | Reasons to stay in care: children and family | 2 | “Now, because I want to ensure my child's future, I'm taking responsibility for myself. I think I can do a lot of things in the future. I realized that this is not the end.” |
|  | Treatment Interruptions | Reasons to stay in care: knowing medication keeps you healthy | 2 | “I want to live, ma'am, that's my motivation. Besides, there are so many other diseases worse than AIDS. I have seen people die of cancer who have suffered a lot, while I realize that if I take my medication regularly, I could die of another disease” |
|  | Viral Load | Community-based viral load | 1 | “Even for the viral load analysis, if you are unable to go to the center, they send a machine to take samples for you.” |
| Descriptive | Diagnosis | Testing because of pregnancy | 13 | “At that time, I was pregnant and they tested me and then they put me on treatment.” |
|  | Diagnosis | Testing due to illness | 10 | “In 2014 I got sick, I was throwing up, I had diarrhea; the next day I went to the hospital for lab exams. I used to always get sick since young age. I even thought it was satanic. I got tested.” |
|  | Diagnosis | Testing in mobile/community clinic | 7 | “The first test I did was in my community during a mobile clinic in 2018. They passed by my home, they did the test for my children, then for me.” |
|  | Diagnosis | Diagnosed during training/activity/for school | 7 | “My first test was done in [clinic]. They were giving out scholarships and had requested all the children to get tested.” |
|  | Psychosocial support | Need for mental health care | 6 | “But I was watching my body and I was like, if I have lesions appearing on my skin, in order to avoid suspicion, I will kill myself” |
|  | Viral Load | Frequency of VL testing | 6 | “But every 3 months I go for my viral load test.” |
|  | Condoms and Lubricants | Using condoms since diagnosis | 5 | “Since I’ve been detected as a PLHIV I’ve always used condoms. I never engage in relations without it, we’ve been advised about it in one of our trainings.” |
|  | Condoms and Lubricants | Refusal/unwillingness to use | 5 | “No. I don't need them.” |
|  | Diagnosis | Testing during other medical care | 3 | “I was a blood donor. I was asked to get tested first. I decided to go to a laboratory to be tested. And I was told that I was HIV positive.” |
|  | Diagnosis | Testing for wedding | 2 | “I was getting ready to get married, and we were asked to get a health certificate. After the exams, they announced this to me.” |
