# Supplementary material for: Drivers of HIV treatment interruption: Early findings from community-led monitoring program in Haiti
Source: PLoS One. 2023 Dec 5;18(12):e0295023. doi: 10.1371/journal.pone.0295023 (PMC10697516; doi:10.1371/journal.pone.0295023)
Supplement: S2 Table — (DOCX) [file pone.0295023.s002.docx]

| **Type** | **Parent Category** | **Child Category** | **N** | **Example quotes** |
| --- | --- | --- | --- | --- |
| Barrier | Staff Attitudes | Rude or unprofessional behavior | 28 | “Yes, I have been humiliated, mistreated, disrespected and denigrated.” |
|  | Staff Attitudes | Stigma and discrimination | 27 | “When you’re sick, they humiliate you, treat you like an animal.” |
|  | Concerns with Medications | Nausea, nightmares, dizziness, fatigue, spitting, rash | 25 | “At first, I was dizzy, I slept very badly. Now I have been given other tablets, it is different. I did not feel any effect.” |
|  | Privacy | Concerns with visibility to other people in clinic | 23 | “The difficulties remain the location of the pharmacy, everyone is looking at us and I don't feel good about myself, the lack of privacy. I am afraid that other people will see me and that the whole community will know that I am infected.” |
|  | Index Testing | Discomfort with/refusal to participate in index testing | 20 | “I was asked to inform [my partner] but I was afraid because I know his personality.” |
|  | Stigma | Discrimination, bullying, and humiliation | 20 | “Sometimes the staff treated us with disdain and even insulted us because of our seropositive status. We were humiliated once they found out about our condition.” |
|  | Stigma | Ostracizing | 19 | “We saw a pretty young lady pass by, I told her that I was interested in this pretty young girl. He told me ‘Stay away from her. She has HIV, she is sick.’ […] The next time I saw this friend, he didn't say hello. When I asked him the reason, he bluntly said, ‘I warned you and you didn't listen to me. We're no longer friends.’ This is how we manage the infected […] you are rejected, that's all.” |
|  | Disclosure | HIV status disclosed by medical staff | 18 | “As soon as you test positive for HIV, the nurse will divulge the information to anyone who crosses her path, to your neighborhood, to your friends if they know them.” |
|  | Privacy | Separation of patients | 18 | “Even in the hospital building, […] at the very beginning of the entrance there is an arrow indicating "HIV patient". This is the space that is made for us. As soon as someone who frequents the hospital sees you going in that direction, you are already identified. And the news is spreading throughout the city.” |
|  | Delivery | Fear of disclosure | 15 | “Because I don't want other people to know that I'm infected, I don't want them to have any idea.” |
|  | Stigma | Losing jobs, access to education | 12 | “At school one time, my classmates went through my bag and found the medication. The principal was informed and expelled me from school.” |
|  | Treatment Interruptions | Stopping treatment due to staff treatment | 6 | “She was standing next to other people who had come to the clinic […] and a security guard spoke loudly to her: ‘You know you are infected, and then you put yourself among the others.’ And this victim [dropped out of treatment], like me.” |
|  | Staff Attitudes | Threatening, aggression | 6 | “And I didn't realize I didn't greet the person at the lab, because I had to do an exam. She exploded to tell me, ‘You come in without greeting me?’ and to add that she was not responsible for my illness.” |
|  | Privacy | Indiscrete medications | 5 | “They get yelled at in the streets, so sometimes they remove the labels. But people tease them even for the sound of the box.” |
|  | Privacy | Counseling in shared spaces | 5 | “There are some places where they receive patients in threes. I could explain something personal to the doctor, but I can't because of the two other people who are close by. Just because we are all PLHIV patients doesn't mean that we don't have a private life, that everyone has the same problem.” |
|  | Treatment Interruptions | Stopping treatment because of fear of disclosure | 5 | “[The clinic] puts us in a room for PLHIV, so once you go in, everyone already knows that you belong to that group. Because of this, I had decided to stop taking my medication at the center.” |
|  | Concerns with Medications | Pills too big | 5 | “Most of the time the pills aren’t easy to swallow. I know some clients who have had to cut them in half. The PrEP pill is huge.” |
|  | Stigma | Homelessness or having to move | 5 | “I was at my parents' house and they chased me away. So I had to go from house to house to find a place to sleep. [I was staying with a friend who] saw that I was taking medication and started to act badly with me. Now I'm somewhere else.” |
|  | Privacy | Colored cards | 4 | “She gave me a card that reflects my situation. And this is where you are stigmatized, as soon as you have that color of the card, you are targeted, we put you in the category. As soon as anyone sees this card in your hands on the premises, you will know you have AIDS.” |
|  | Privacy | ARV-only clinics | 4 | “It would be better if we had access to other services, because it doesn't make sense to me to be limited to only offering HIV drugs. You may have another health problem that requires treatment at the center, as soon as you see me, I am automatically linked to AIDS, they are about to give me the bag containing the drugs.” |
|  | Concerns with Medications | Weight gain/weight loss | 4 | “I have a problem with that. the medications have not too interesting effects. I am getting very fat and drinking a lot more than usual.” |
|  | Stigma | Self-stigmatization | 4 | “I didn't feel comfortable, I didn't feel good. I was judging myself very badly. […] I was living a bad life and I felt guilty, I had regrets.” |
|  | Transfers | Denied transfer | 4 | “Everyone needs numbers, [the clinic] wants to justify providing the service to a lot of PLHIV, they are not going to [agree to a transfer].” |
|  | Stigma | Violence | 3 | “A nephew of mine threatened me, he said if he ever tests positive for the aids, he will behead my children. He said he will make sure to erase me from the family.” |
|  | Transfers | Want/received transfer due to availability of medications or services | 3 | “There were no other medicines at [my first clinic]. It was at [the second clinic] that I could find other medicines that did not bother me.” |
|  | Disclosure | Refusing care from fear of disclosure | 2 | “I used to go to [that clinic] but I stopped going because too many people would know my business.” |
|  | Disclosure | Disclosure due to patient visibility | 2 | “I met someone at the hospital that my parents knew. Then he went to question my family members to find out the reason for my visit to the hospital. Since then, I have decided not to go there anymore.” |
|  | Privacy | Lack of interest from staff in protecting privacy | 2 | “However, when I talk to them about the way we are welcomed in the room, they tell me that it is not up to them, it is the responsibility of the administration to manage this problem.” |
|  | Treatment Interruptions | Stopping treatment because of fear of medical care | 2 | “A lot of KP are scared of hospitals. Sometimes, they would set an appointment, and not show up. Some of them only feel comfortable in their communities.” |
|  | Staff Attitudes | Denial of care | 2 | “I had a toothache once and needed it to be taken care of, [the doctor] denied me the care. She said because I [transferred out of] the hospital, somehow they misplaced my file. She is still punishing me because I left.” |
|  | Transfers | No way to report problems | 2 | “As there is no place to report those bad behaviors towards us, I have to [transfer] elsewhere.” |
|  | Treatment Interruptions | Stopping treatment because of feeling unsafe | 1 | “No, [feeling unsafe in the hospital] is one of the reasons I've lost motivation. I don't pick up my medication anymore” |
|  | Transfers | Transfer to private sector | 1 | “Sometimes the tests are available at the hospital but the doctor has his own places he wants to send you. Sometimes the tests are not expensive at the hospital and then you are sent somewhere else in the private sector which is very expensive” |
| Enabler | Staff Attitudes | Positive or neutral experiences | 31 | “I am about 14 years since receiving the service that my case requires at [this clinic], the service is impeccable. I am given medication every 6 months, and when I go the service is always good for me.” |
|  | Privacy | Received discreet services | 13 | “They keep my condition secret, some of them even know that I’m gay. Everything remains personal and discreet.” |
|  | Disclosure | No concerns about disclosure | 1 | “Maybe other people seeing a patient leave the center can spread the information, but I don't think an employee would disclose a patient's confidential information to others.” |
| Descriptive | Index Testing | Participated in index testing | 22 | “I was pregnant while I was in the hospital, they did the test for me, they found me positive, they told me to come with my husband. I brought him and they did the test for him.” |
|  | Disclosure | Decision to not disclose to family or partners | 17 | “If my family knows, they will abandon me. Maybe my mother could handle me, but she would die.” |
|  | Transfers | Moving between facilities without approved transfer | 9 | “So, as often as I am not satisfied in a center, I go elsewhere. And this without taking a transfer, because it is my life that is at stake.” |
|  | Transfers | Want/received transfer for discrimination or bad treatment | 7 | “Yes, there is discrimination, that's what I saw and that's why I asked for the transfer.” |
|  | Disclosure | Decision to disclose to family or partners | 5 | “I asked him if he knows somebody that have HIV in their blood what would he do. He said that it’s not the end of the world, just need to be careful and help that person to move on. He saw that I was sad he asked me what was wrong. I went in my bag and showed him the result. He said ‘No need to feel sorry, life is not over. Just accept and continue living.’” |
|  | Concerns with Medications | No concerns | 4 | “A white pill in the morning and a pink one in the evening. They don't bother me. The first one they gave me before didn't bother me either.” |
|  | Disclosure | Consequences of disclosure | 3 | “I don't have a business and my family disowned me as soon as they found out I was on ARV.” |
|  | Transfers | Transferred by clinic | 3 | “I was in a center but they didn't seem to have a plan to take care of sick people. They sent me to another bigger hospital that asked me for my passport.” |
|  | Index Testing | Was not asked to participate | 1 | “The test was positive but I was not asked to come with my partner” |
