# Supplementary material for: Drivers of HIV treatment interruption: Early findings from community-led monitoring program in Haiti
Source: PLoS One. 2023 Dec 5;18(12):e0295023. doi: 10.1371/journal.pone.0295023 (PMC10697516; doi:10.1371/journal.pone.0295023)
Supplement: S3 Table — (DOCX) [file pone.0295023.s003.docx]

| **Type** | **Parent Category** | **Child Category** | **N** | **Example quotes** |
| --- | --- | --- | --- | --- |
| Barrier | Wait Times | Long wait time | 27 | “At [this clinic], you could go very early in the morning, you sleep there, you wake up there, yet can never go. You could arrive at the hospital around 6 o'clock in the morning and be able to return home at 4-5 pm. That’s why I asked for a transfer.” |
|  | Stock-outs | Medication unavailable | 11 | “The best thing is that the center should not be out of stock. If we travel to get the drugs, the center must be able to serve us, especially those who come from far away.” |
|  | Delivery | Delivery not available | 8 | “But, if I could find a delivery service, that would be nice. If we could have a trained agent who could bring them to my house, I wouldn't have to go anywhere.” |
|  | Condoms and Lubricants | Not available | 7 | “I always find condoms but I never find lubricant. When I ask, they never have any to give away.” |
|  | Prescription Length | Refills 2 month or less | 6 | “I have to go get them on a monthly basis, but if I could get them for a duration of 3 months it would be more convenient.” |
|  | Treatment Interruptions | Stopping treatment due to challenges getting to clinic | 6 | “I was 14 months in the Dominican Republic, I ran out of medications. I could not return yet and I was 2 months old without taking medication. Finally, I went to Haiti to continue with the treatment because I became weak.” |
|  | Wait Times | Faster/unequal service for certain patients | 6 | “They make a selection. If you have money, they will serve you as quickly as possible, but the category of those who have no money, like me, they make you suffer.” |
|  | Delivery | Bad coordination | 5 | “Yes, but every time I call the agent, it's the same story. ‘He will come, he will come,’ and he never came.” |
|  | Stock-outs | Expired medications | 5 | “They can tell me that there is no new stock, so they deliver you a drug that is expiring.” |
|  | Travel | Going to farther clinic to avoid being seen by community | 4 | “I’d rather go far, so I can live my life in peace without fearing someone would talk about my business. I’m often in [this area], I went to school there, so I get my care far away so I can keep my confidentiality.” |
|  | Travel | Concerns about travel distance | 4 | “It’s not about getting sick, sometimes, the distance is difficult.” |
|  | Prescription Length | Offered long stock but refused | 4 | “When I was offered it, they told me to take a test and offered it for a period of 6 months. I didn’t want it for so long. I didn’t want to raise suspicions at home.” |
|  | Support Groups | Barriers to participating in groups | 3 | “Yes, they offered a support group but I have no time because of my children. If I have to be out they have to stay with the neighbors.” |
|  | KP Services | Want more general services in KP clinic | 3 | “If the clinic also offered other services it would be the best alternative for the KP.” |
|  | KP Services | Barriers to care for KP | 3 | “KP are discriminated against almost everywhere. They are very apprehensive about the hospital.” |
|  | Prescription Length | Rx length depends on stock | 3 | “I was first tested at [one clinic], I was given 5 to 6 pills the next time I was given 2. I came back and was told that there was no more. I went to [another clinic] and was given 3 jars for 8 months.” |
|  | Delivery | Preference to go to clinic: other services | 2 | “When one [picks up medications] you can take the opportunity to check my blood pressure. It’s better than when they bring it home. If you have another problem such as a headache or anything else, you can tell the staff. That does not happen when you bring.” |
|  | Treatment Interruptions | Stopping treatment due to wait time | 2 | “They waste a lot of time at the hospital and they decide not to return. We lose a lot of people because of how much time the doctors take to attend to them.” |
|  | Wait Times | Need to arrive early | 2 | “The most important thing in the hospital is that it's first come first served, so you have to make the strategy and wake up early.” |
|  | Wait Times | Clinicians starting late or working slowly | 2 | “People reach very early at the hospital to get service, but the doctors sometimes don't arrive until 10:00am.” |
|  | Support Groups | Want services not offered | 1 | “What I would like to see improvements in is to expand the space and work with other groups so that we can meet, socialize, organize outings, go to the sea. So that we can have fun together. We never go anywhere.” |
|  | KP Services | No services available | 1 | “They don’t have a program for gay men.” |
|  | KP Services | Lack of trans services/hormone therapy | 1 | “I’d advocate for centers exclusive to trans. Hormones are important. […] A lot of trans people are living in HIV and they don’t want to go where they treat gay people because when you’re trans you get outcasted.” |
|  | Stock-outs | Shortened prescriptions | 1 | “I use to attend to [the hospital] but sometimes they reduce the amount of medication to avoid external distribution by some.” |
|  | Stock-outs | Preferential dispensing | 1 | “These days, they’re lacking and when their stock is low, they only give it to their friends. If you have no contact, you don’t get any.” |
|  | Travel | Going to farther clinic because of safety concerns | 1 | “I used to go to the one in Verette but I’m too scared of the road now and so I don’t go there anymore.” |
|  | Travel | Going to farther clinic at clinic request | 1 | “When I asked him for advice he said the best would be to go to Port-au-Prince for treatment. But it was difficult going to Port-au-Prince.” |
|  | Wait Times | Wait times interfering with work | 1 | “This patient works in a factory. To save time, she paid 750 gourdes in motorcycle fees to get there. She explained the situation and asked for the medication so she could return to work. It was ignored. They could bring it to her at work, but the lady had to waste all that time on medicine.” |
| Enabler | Prescription Length | Refills 3+ months | 31 | “They used to give it for 3 months but now they give it to me for 6 months.” |
|  | Wait Times | No concerns | 12 | “I don't waste time, it depends on me. If I explain that I am in a rush, I am served immediately.” |
|  | Delivery | Delivery improving retention, reengagement in care, and reducing LTFU | 11 | “Sometimes I know how to be negligent and I don't go [to the clinic]. But since they bring it to me, I don't neglect anymore. It's already been 1 year since they brought it to me.” |
|  | Stock-outs | No stock-outs | 11 | “There is always medication, it is not difficult for me to find.” |
|  | Delivery | Good practices | 9 | “The agent gave me the bag and said he was leaving me a gift. Since I was talking to a friend who was interested in knowing what gift and wanted me to share it with her, the agent quickly realized that he had to change that the gift was not mine, I had to keep it for him and he would come by to pick it up.” |
|  | Condoms and Lubricants | Available | 8 | “They give me condoms and lubricants, each time I go they always give me.” |
|  | Delivery | Delivery saving patients time | 5 | “Now they have a trick, they bring it to you, they can come with the machine to do the viral load. So now I meet them halfway to pick up the medicine, it's better for me, I don't lose time if I have to go to the market” |
|  | Delivery | Alternative ideas | 5 | “What I want to say in regards to the clients, is that some of them live very far away from the site. We need to think about an itinerary that’s more practical for the peers. And if there could be other pick up locations closer to the communities, that could also be useful.” |
|  | Wait Times | Appointment system | 4 | “At [the clinic] they give you an appointment card. If you are not present at the appointments they call you if they still can't reach you they send someone directly to your home.” |
|  | Delivery | Friends picking up medicines | 3 | “No there are times I cannot go [to the clinic], I cannot leave the children alone. So my friend brings it to me sometimes.” |
|  | KP Services | Importance of peer companions / KP or PLHIV staff | 3 | “If the person providing the service was LGBT or had the virus, the patient would feel much more confident. People are not open-minded like you. You should feel confident to explain to the person the difficulties you are facing.” |
| Descriptive | Delivery | Preference to go to clinic: unspecified | 9 | “Getting my medication is the best option, I don't like it being delivered.” |
|  | Delivery | Preference to go to clinic: not needing help | 4 | “I will get my medication. It's a personal thing, I won't allow someone else to do it for me.” |
