# Supplementary material for: Drivers of HIV treatment interruption: Early findings from community-led monitoring program in Haiti
Source: PLoS One. 2023 Dec 5;18(12):e0295023. doi: 10.1371/journal.pone.0295023 (PMC10697516; doi:10.1371/journal.pone.0295023)
Supplement: S4 Table — (DOCX) [file pone.0295023.s004.docx]

| **Type** | **Parent Category** | **Child Category** | **N** | **Example quotes** |
| --- | --- | --- | --- | --- |
| Barrier | Employment | Desire for employment | 13 | “Having a business, so when I need to take medicine I can buy some food. If I can find a little work when I need money I can manage on my own. I'm someone who likes to do business, marketing, that I can do.” |
|  | Psychosocial support | Need for food or economic support | 12 | “I also have a 16-year-old child who is infected, and every time he says he doesn't feel well, I feel like I'm the one who is infected. Every time he takes the medicine, he tells me ‘But mom, I’m hungry. I can't take the medicine without food.’” |
|  | Cost | Not getting services they want/need because of transport costs | 8 | “I take a moto ride to go [to the clinic] and my son goes with me. Every trip costs us 1,000 gourdes and we don’t always have the means to go.” |
|  | Cost | Need food to take medicines | 8 | “The issue of finding food is a huge challenge. A lot of patients complain that if they don’t eat, they cannot drink [the medicine].” |
|  | Cost | Costs of other healthcare services | 7 | “What I would like is just like the HIV treatment is free, some analyses are free but other analyzes like heart exam, sonography you need to pay for them.” |
|  | Concerns with Medications | Side effects due to not eating | 7 | “I get short of breath and sometimes I get dizzy if I don't eat, and the pills are big, you have to eat. Before, we used to accompany the medication with a small food kit, we don't do that anymore. If a person who doesn't work, who doesn't earn anything, has to take these drugs, they have to find something to eat.” |
|  | Employment | Losing job / facing stigma due to HIV status | 5 | “Even if you get a job as a maid, if the person you're working with knows you're infected with the virus, that's it.” |
|  | Cost | Need for financial support (support for children, money, housing) | 4 | “What I would need as improvement is a consideration for my children. I don’t have the means to support them.” |
|  | Cost | Transport fee not available | 4 | “When I ask for money for transportation fee, they said it would be available at 2 o’clock. I have to beg in the streets so that someone gives me 50 gourdes to go home.” |
|  | Cost | Incentives - unavailable | 3 | “They said they did it before but not anymore. Now if they give you 100 HTG they have to wait 3 hrs or 4 hrs to get it.” |
|  | Treatment Interruptions | Stopping treatment because of side effects or no food | 3 | “I stopped taking the pills because every time I take them I get dizzy, tired and feel sleepy besides I don't eat well.” |
| Enabler | Cost | Incentives - available | 9 | “After that it’s difficult to convince anyone to come back for treatment. I sometimes need to give them incentives: USB keys, radios, just to motivate them for an educational session or for treatment.” |
|  | Cost | HIV medicines/services are free | 3 | “For PLHIV, they just find medicine and consultation free of charge.” |
|  | Delivery | Delivery saving patients money | 3 | “Sometimes I could not afford to pay for the transportation to go and get [medications]. That's why I was negligent. Now they have realized that we have difficulties and they have started to bring it to us.” |
|  | Employment | HIV support jobs for PLHIV | 2 | “It would be good to install a vocational school for gay men to avoid having to face discrimination.” |
