# Supplementary figures and images for: Drivers of HIV treatment interruption: Early findings from community-led monitoring program in Haiti
Source: PLoS One. 2023 Dec 5;18(12):e0295023. doi: 10.1371/journal.pone.0295023 (PMC10697516; doi:10.1371/journal.pone.0295023)

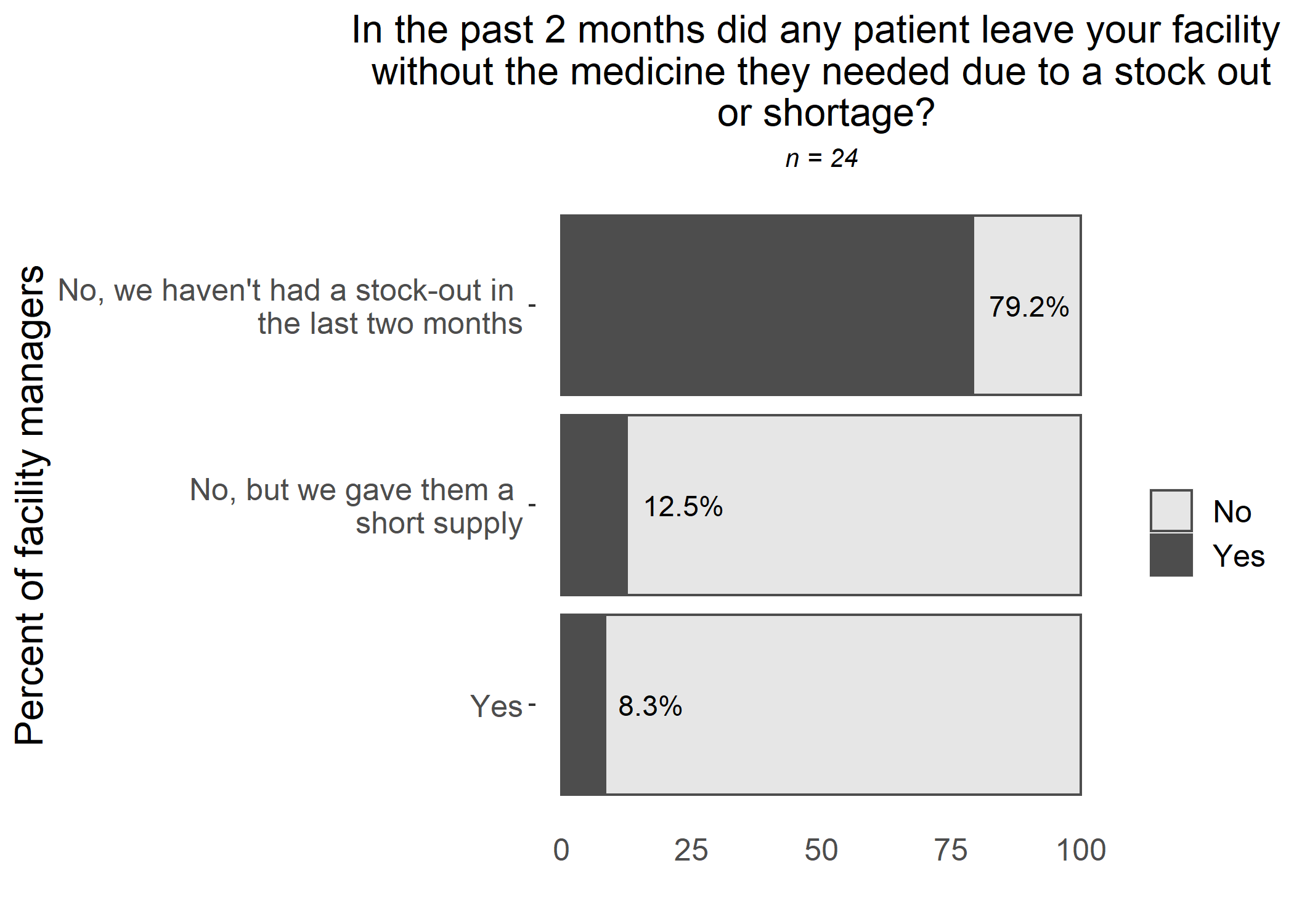

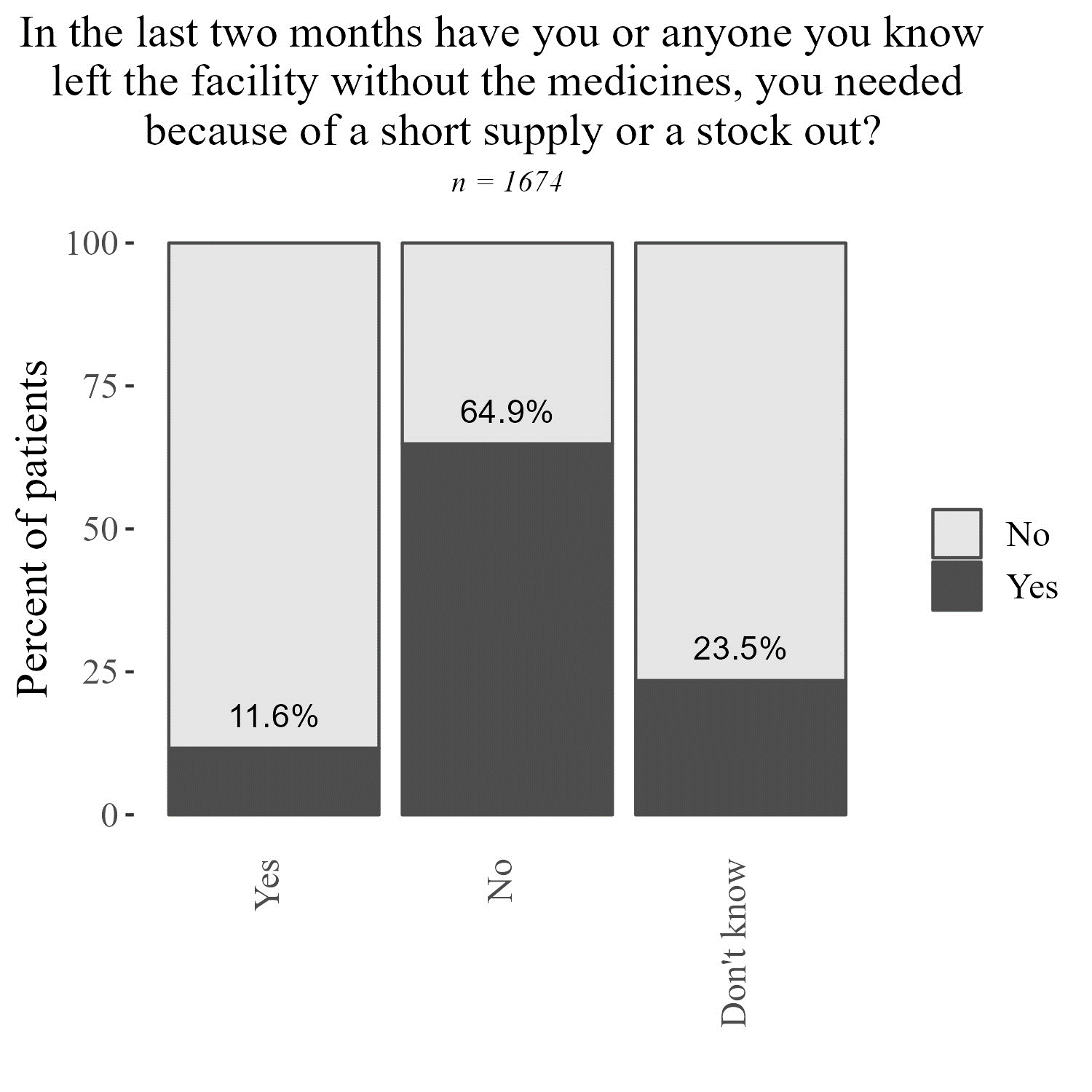

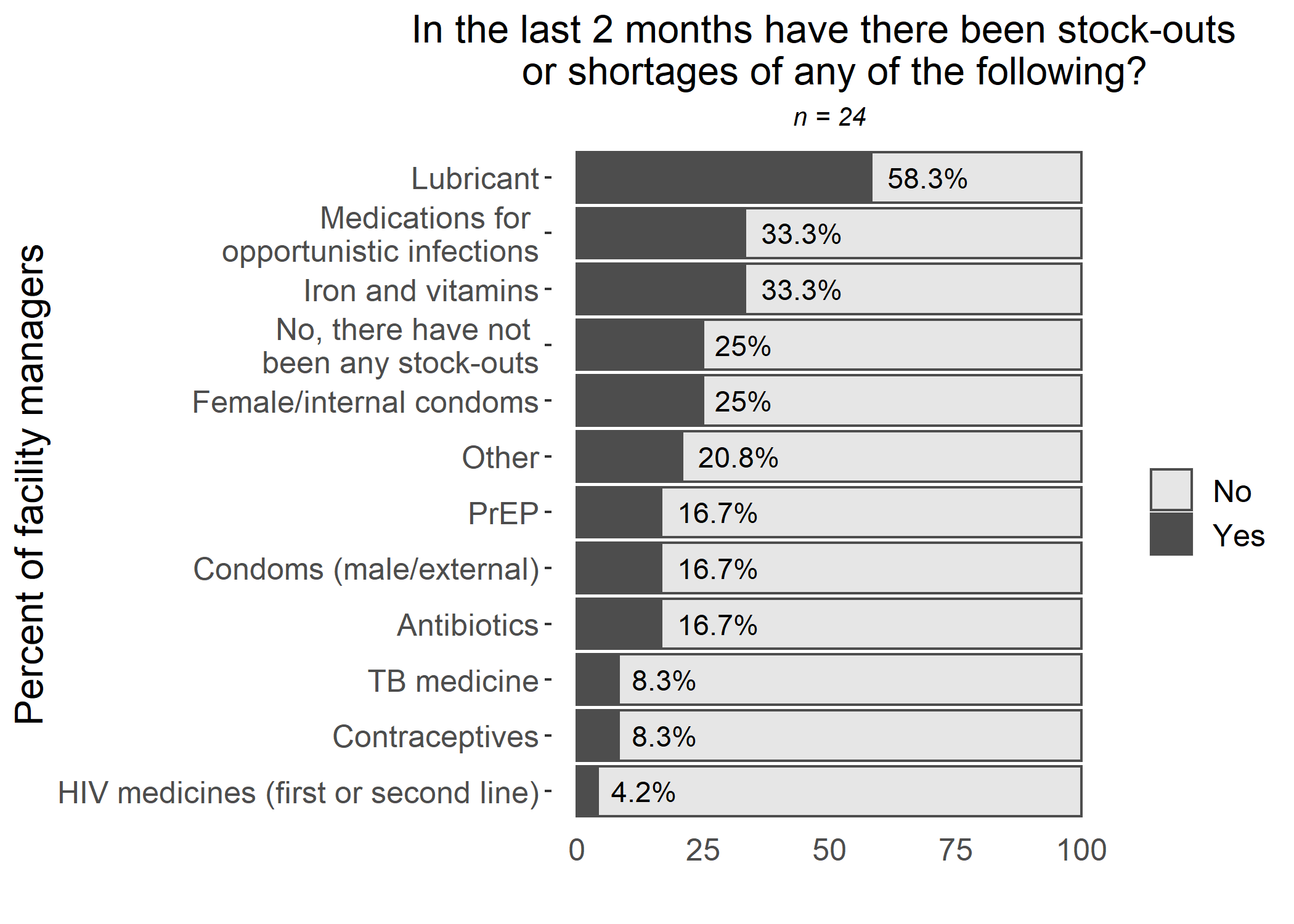

Supplement: S1 Fig — Sample size varies due to skip logic and questions not asked in all rounds of data collection. (DOCX) [file pone.0295023.s008.docx]

| 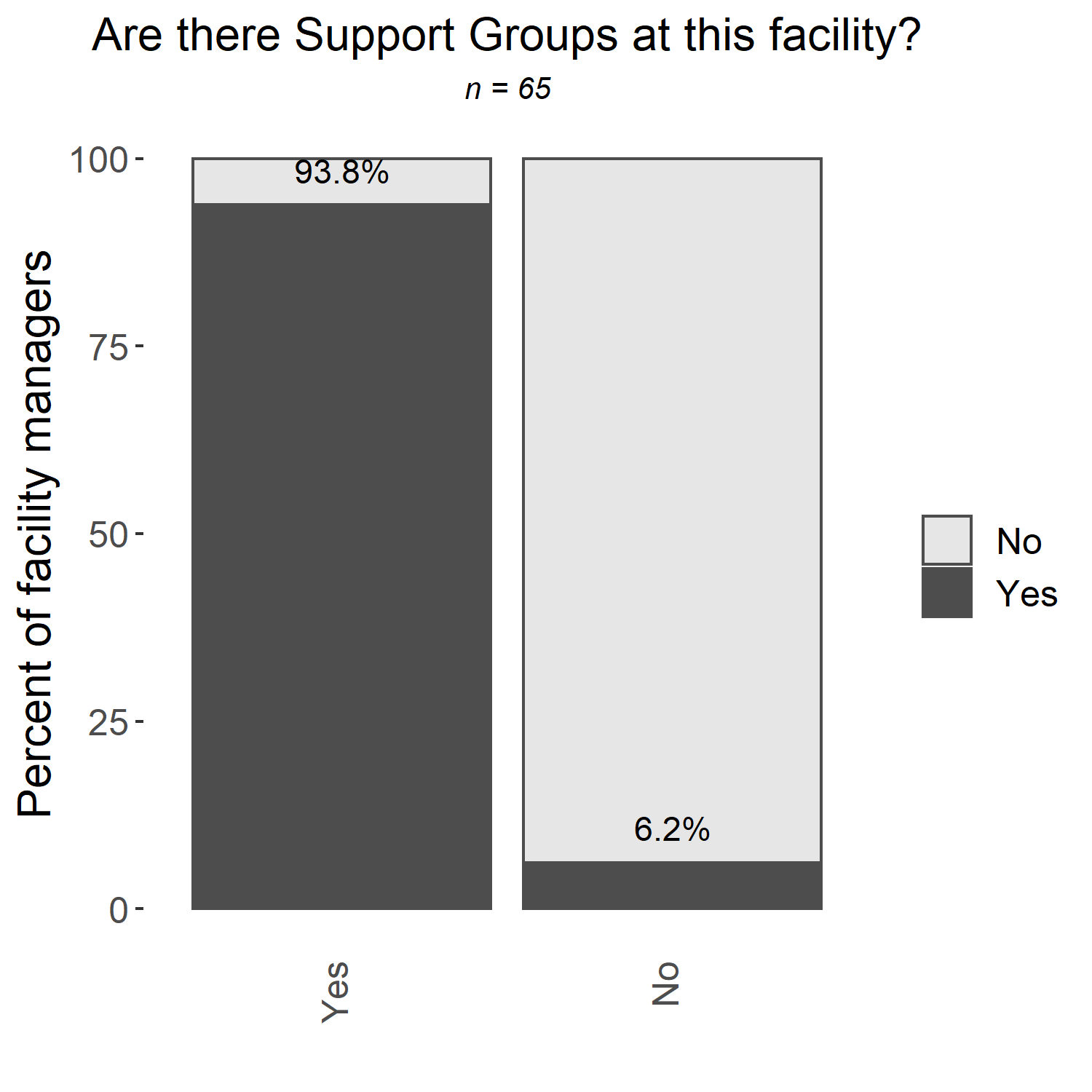 | 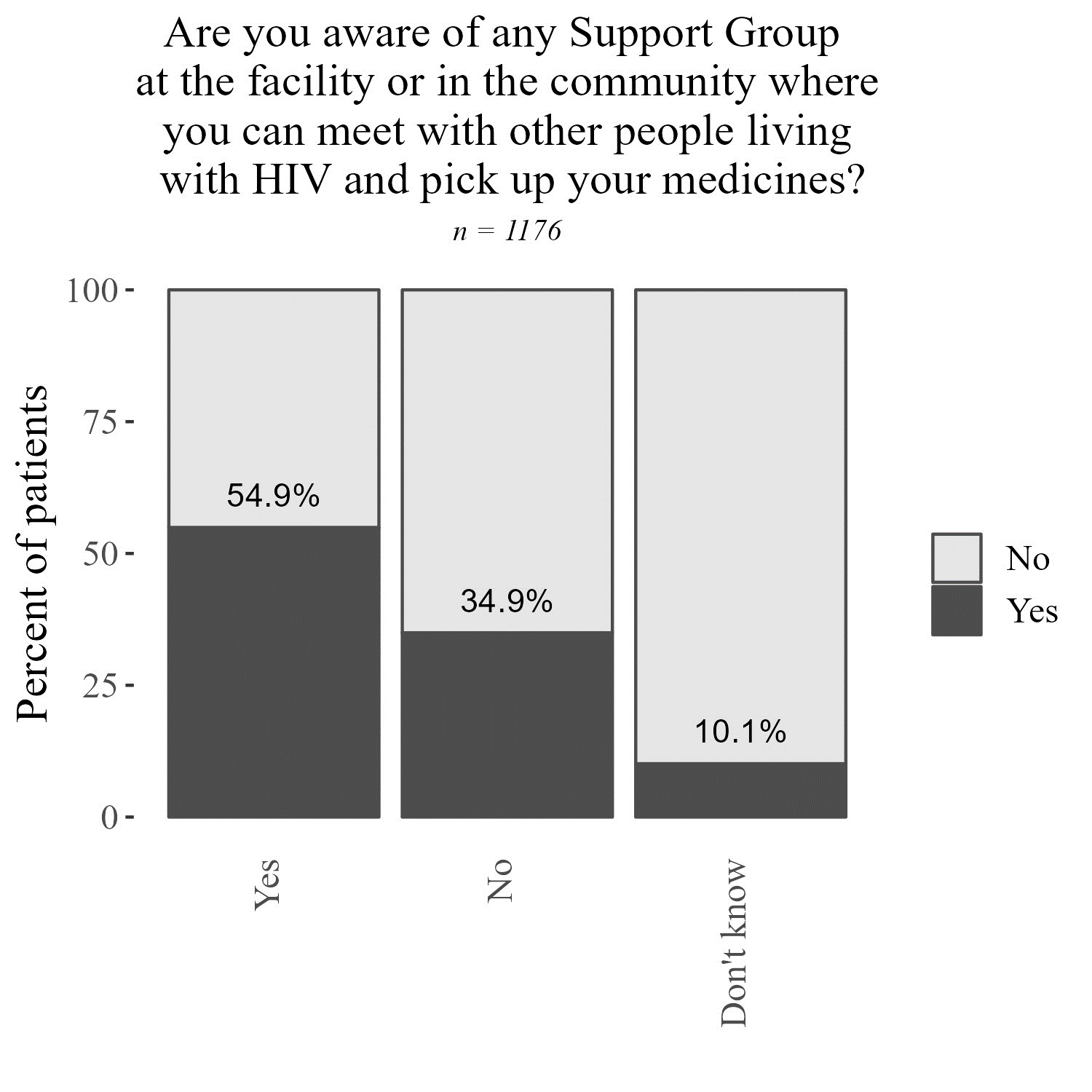 |
| --- | --- |
| 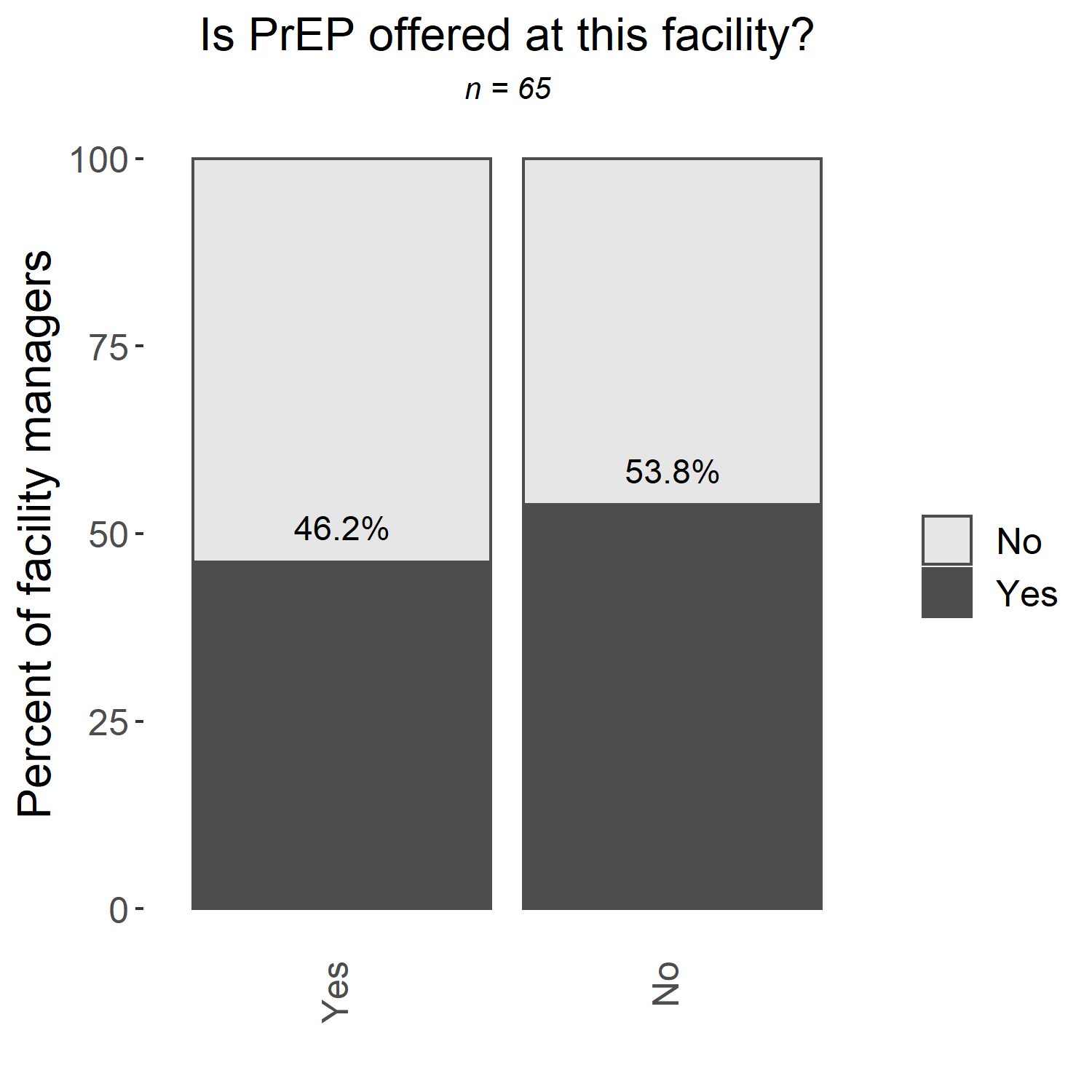 | 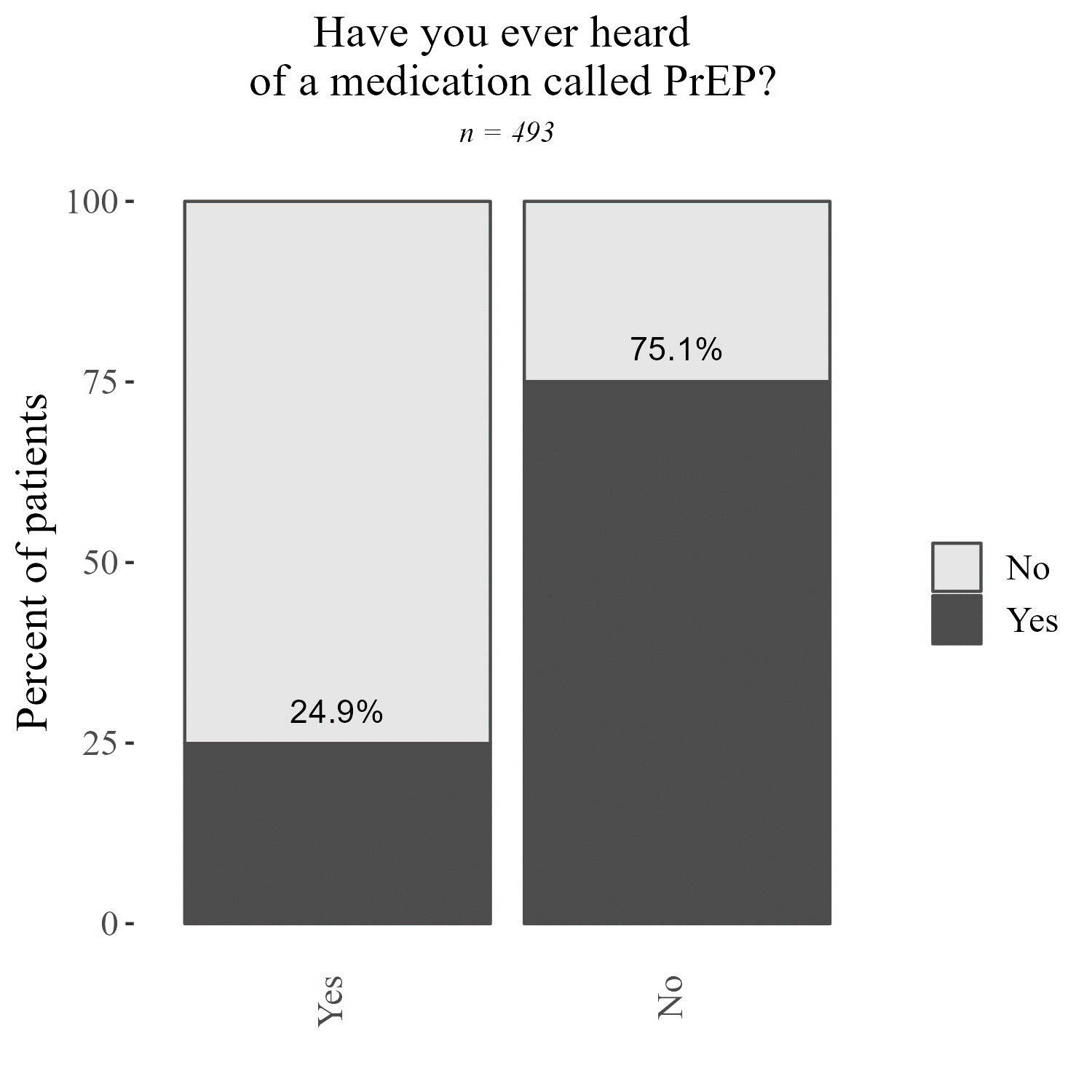 |

Supplement: S2 Fig — Sample size varies due to skip logic and questions not asked in all rounds of data collection. (DOCX) [file pone.0295023.s009.docx]

| 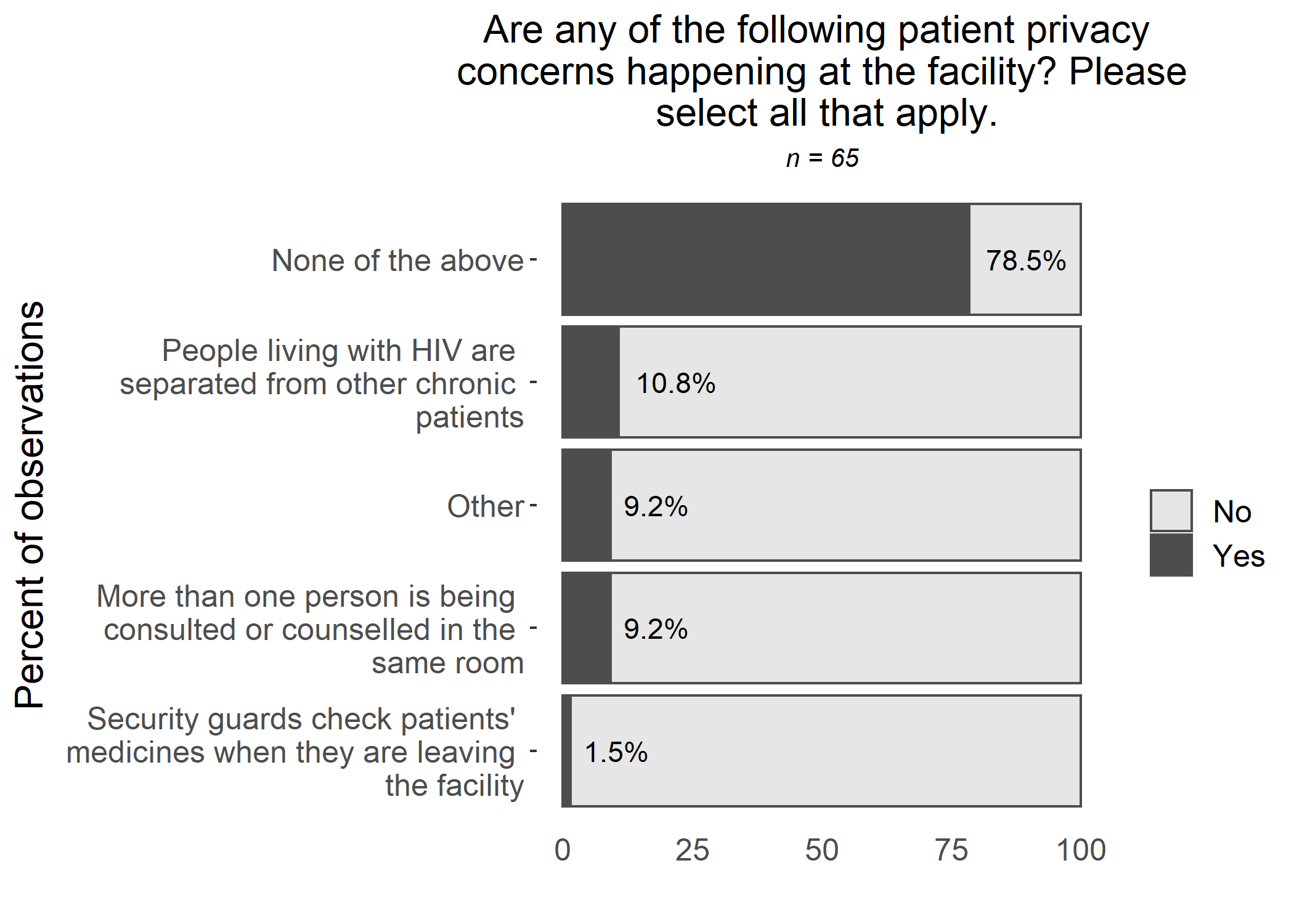 | 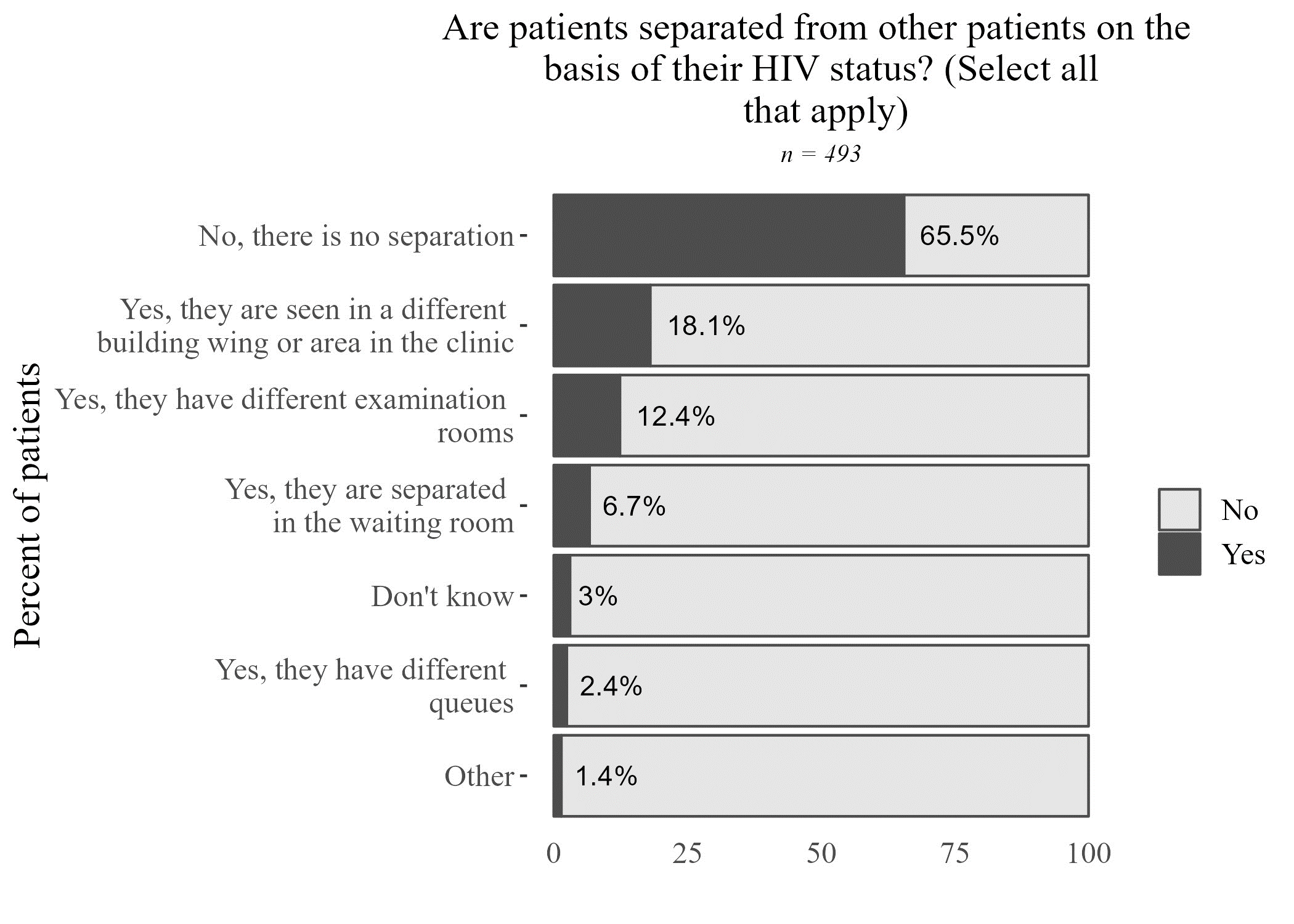 |
| --- | --- |

Supplement: S3 Fig — Sample size varies due to skip logic and questions not asked in all rounds of data collection. (DOCX) [file pone.0295023.s010.docx]

| 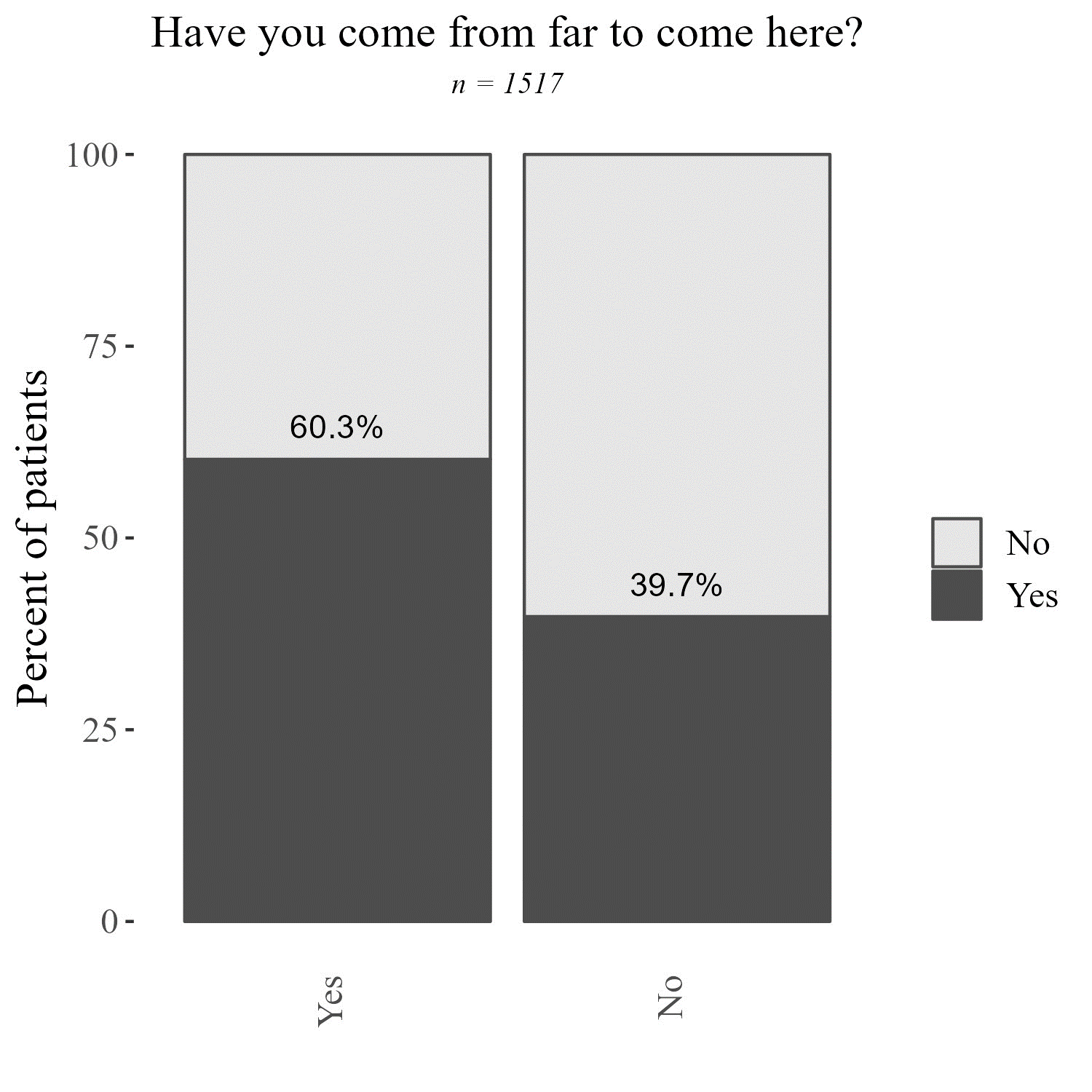 | 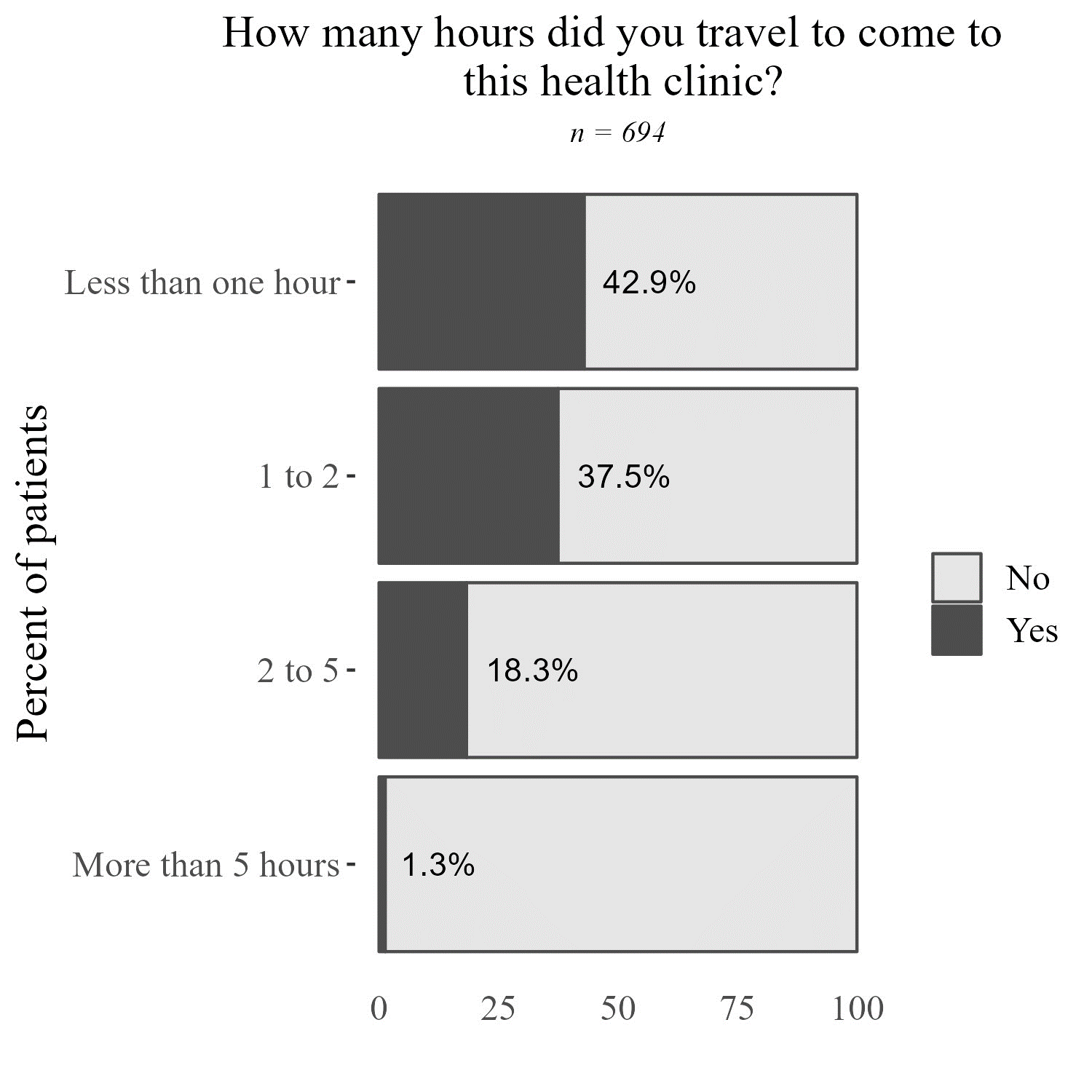 |
| --- | --- |

Supplement: S4 Fig — Sample size varies due to skip logic and questions not asked in all rounds of data collection. (DOCX) [file pone.0295023.s011.docx]

| 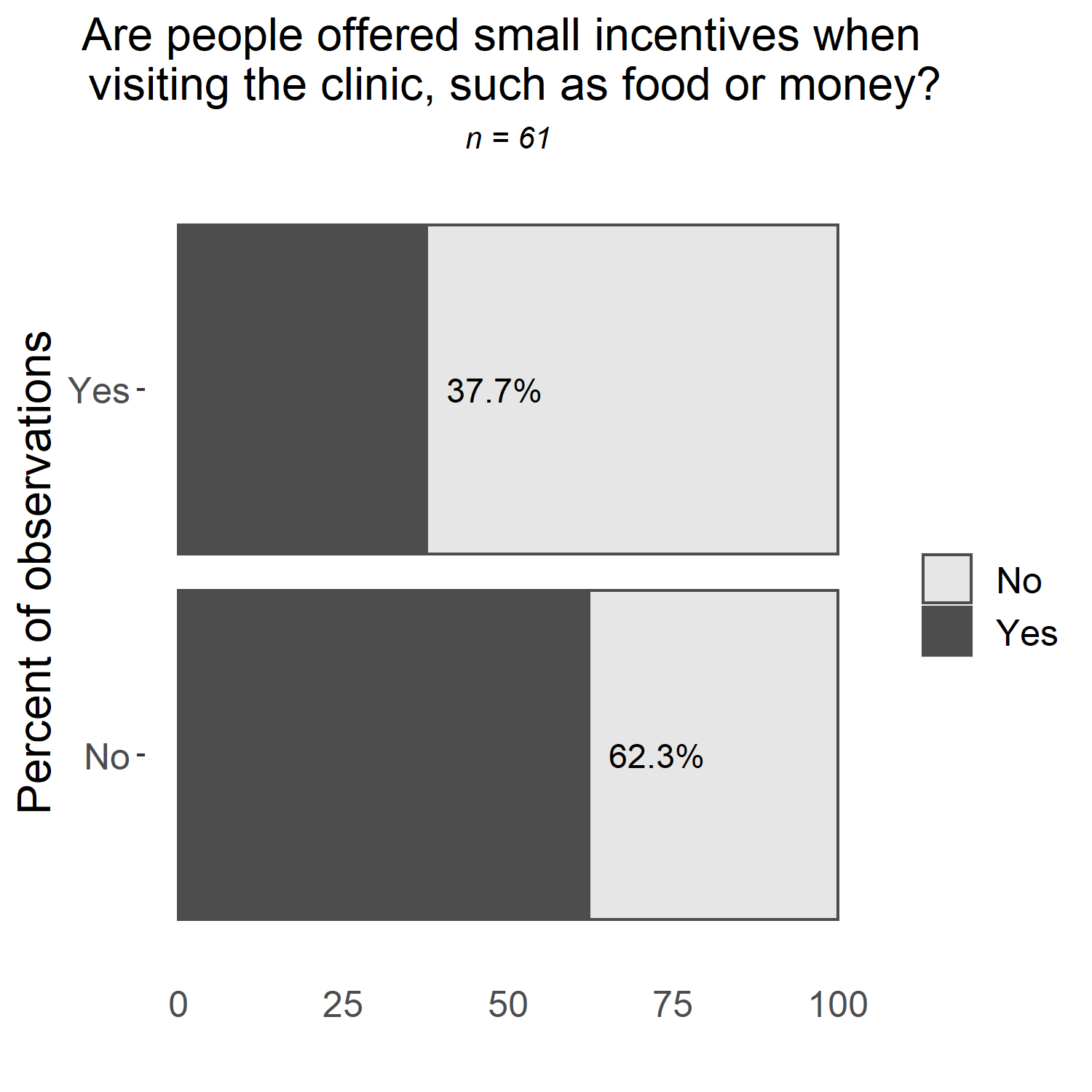 | 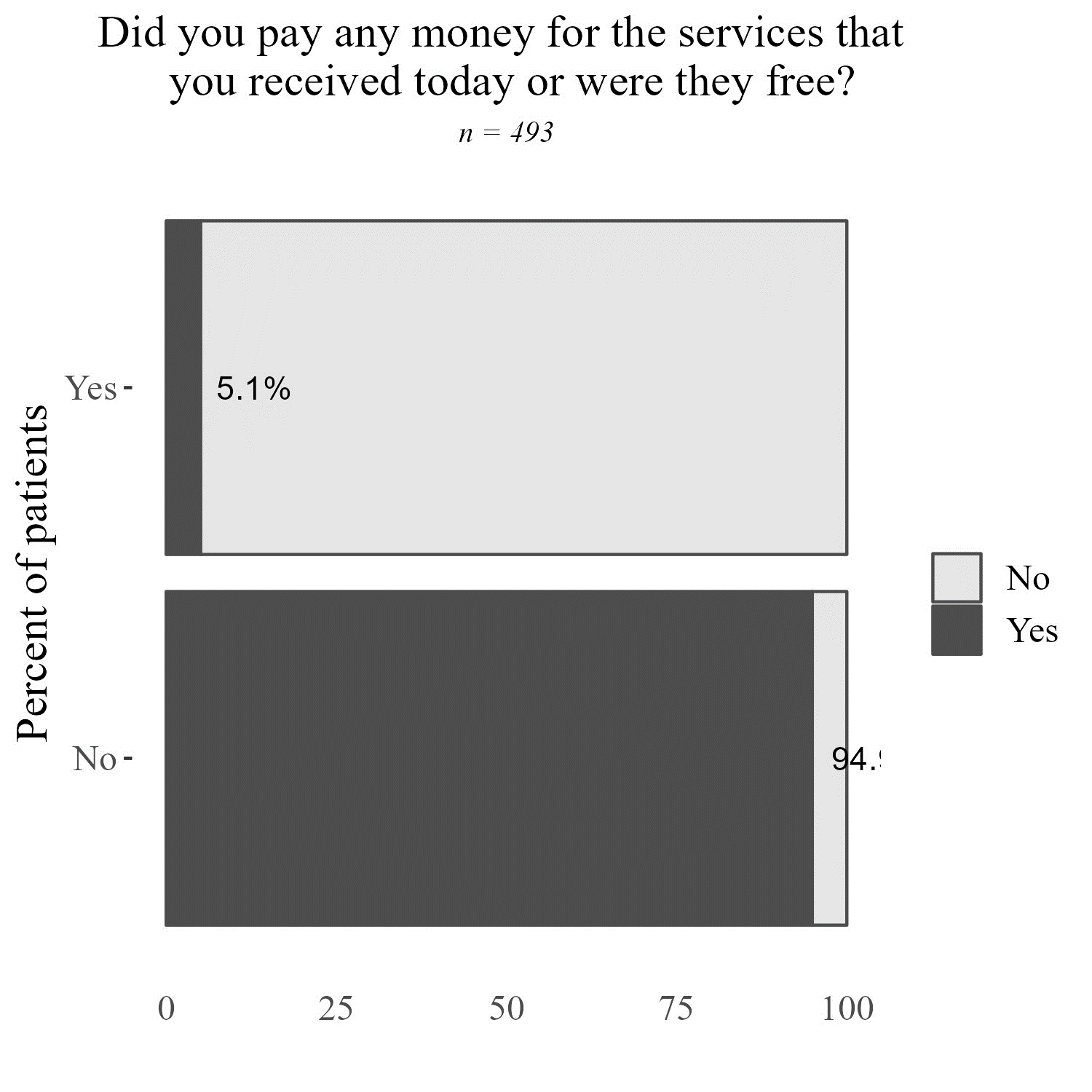 |
| --- | --- |
| 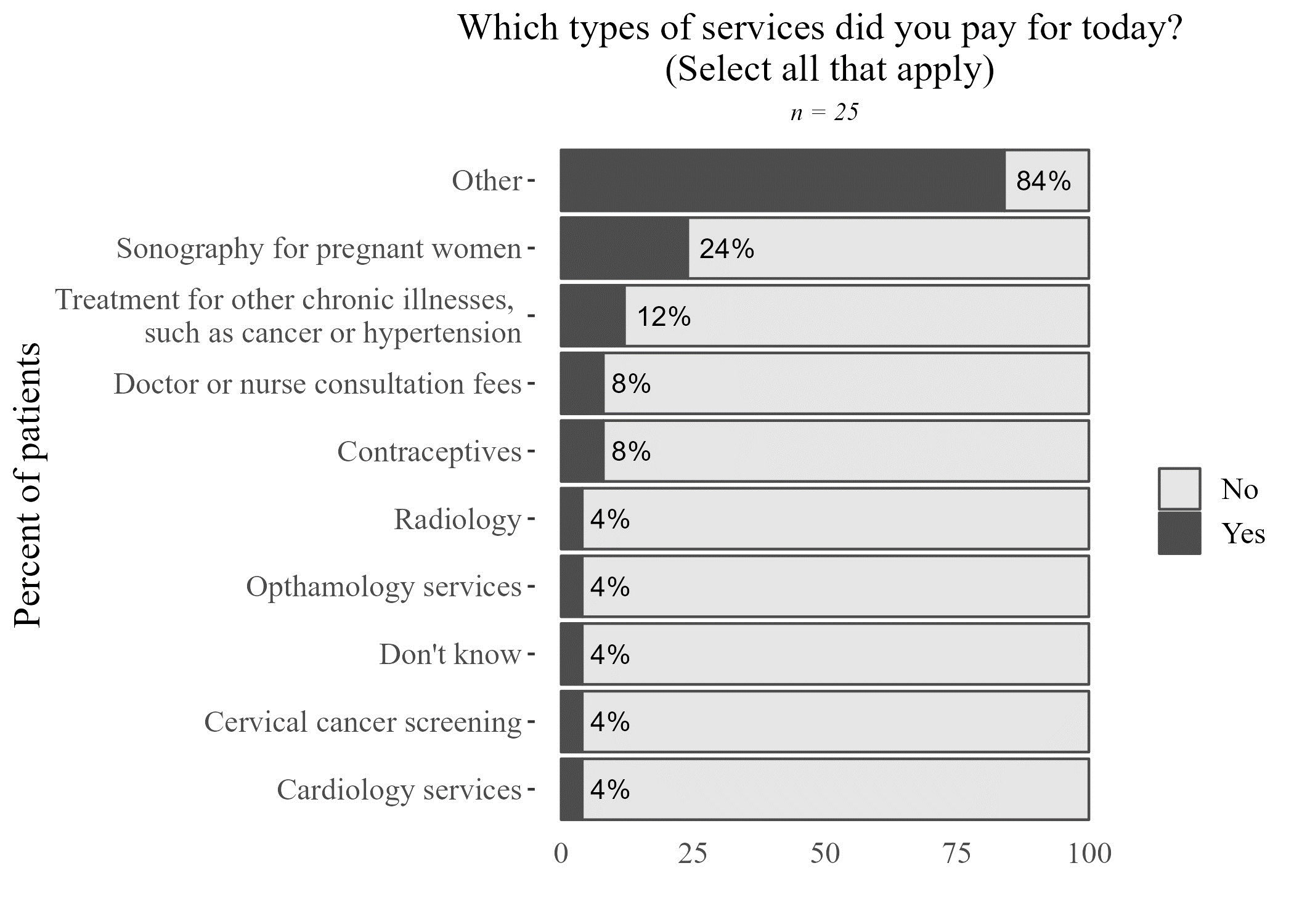 | 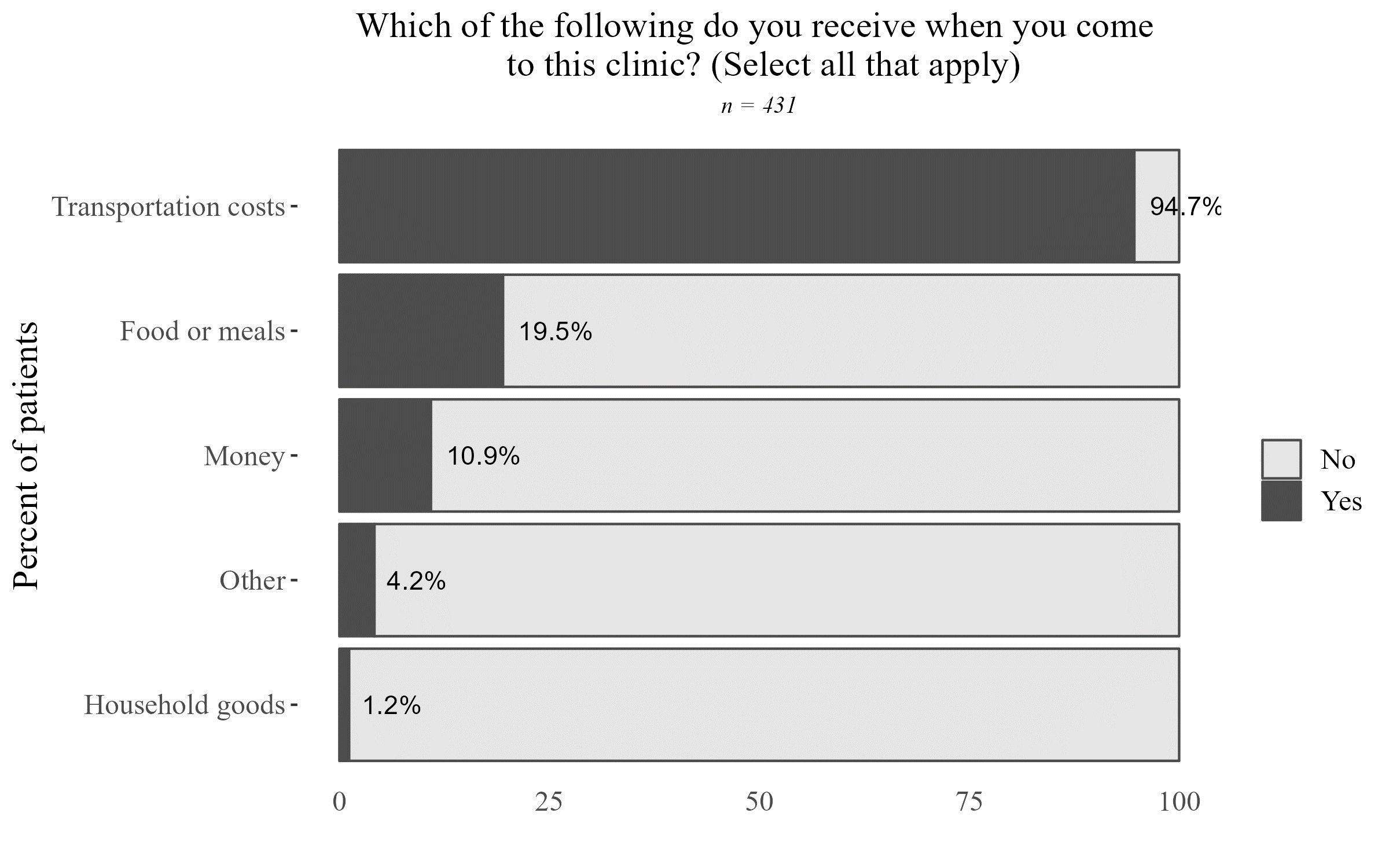 |

Supplement: S5 Fig — Sample size varies due to skip logic and questions not asked in all rounds of data collection. (DOCX) [file pone.0295023.s012.docx]
